# Redox‐dependent binding and conformational equilibria govern the fluorescence decay of NAD(P)H in living cells

**DOI:** 10.1002/1873-3468.70125

**Published:** 2025-07-25

**Authors:** Thomas S. Blacker, Nimit Mistry, Nicoletta Plotegher, Elizabeth R. Westbrook, Michael D. E. Sewell, John Carroll, Gyorgy Szabadkai, Angus J. Bain, Michael R. Duchen

**Affiliations:** ^1^ Research Department of Structural & Molecular Biology University College London UK; ^2^ Research Department of Cell & Developmental Biology University College London UK; ^3^ Department of Physics & Astronomy University College London UK

**Keywords:** FLIM, fluorescence, fluorescence anisotropy, fluorescence lifetime, metabolism, microscopy, NAD(P)H, NADH, NADPH

## Abstract

When probed in living systems using fluorescence lifetime imaging microscopy (FLIM), the emission from reduced nicotinamide adenine dinucleotide (NADH) and its phosphorylated form NADPH have shown promise as sensitive intrinsic reporters of metabolism. However, an incomplete understanding of the biochemical processes controlling their fluorescence decay makes it difficult to draw unambiguous conclusions. Here, we utilised time‐resolved fluorescence anisotropy imaging to identify multiple enzyme binding configurations associated with lifetimes both longer and shorter than unbound NAD(P)H. FLIM, combined with mathematical and computational modelling, revealed that the redox states of the NAD and NADP pools control the steady‐state equilibrium of binding configurations, which in turn determines the observed fluorescence decay. This knowledge will be foundational to developing the accurate interpretation of NAD(P)H FLIM.

## Graphical abstract

In this work, we reveal how different enzyme binding configurations influence the fluorescence decay of NAD(P)H in live cells using time‐resolved anisotropy imaging and fluorescence lifetime imaging microscopy (FLIM). Mathematical modelling shows that the redox states of the NAD and NADP pools govern these configurations, shaping their fluorescence lifetimes. This insight advances the interpretation of NAD(P)H FLIM as a metabolic reporter in living systems.

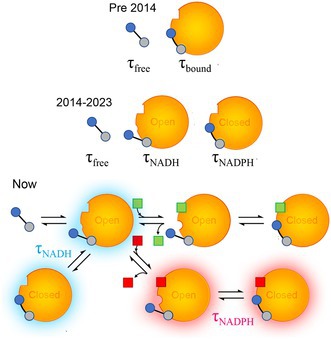

## Abbreviations

**FLIM**, fluorescence lifetime imaging microscopy

**IRF**, instrument response function

**MSC**, Mesenchymal stem cell

**NAD(P)H**, combined NADH and NADPH

**NAD**, combined (reduced and oxidised) nicotinamide adenine dinucleotide pool

**NAD+**, oxidised nicotinamide adenine dinucleotide

**NADH**, reduced nicotinamide adenine dinucleotide

**NADP**, combined (reduced and oxidised) nicotinamide adenine dinucleotide phosphate pool

**NADP+**, oxidised nicotinamide adenine dinucleotide phosphate

**NADPH**, reduced nicotinamide adenine dinucleotide phosphate

**S(ox)**, oxidised enzyme substrate

**S(red)**, reduced enzyme substrate

**TCSPC**, time‐correlated single photon counting

**trFAIM**, time‐resolved fluorescence anisotropy imaging

The pools of nicotinamide adenine dinucleotide (NAD) and its phosphorylated analogue NADP play essential roles in cellular metabolism, ferrying electrons to and from redox reactions crucial to energy production, biosynthesis, antioxidant defence and cellular homeostasis [1]. Preservation of both cofactors is linked to healthy ageing [2, 3], and their disruption is implicated in various diseases [4], making them promising targets for therapeutic intervention [5]. In their reduced (electron carrying) forms, NADH and NADPH are intrinsically fluorescent, and this is lost upon oxidation to NAD+ and NADP+. As the fluorescence spectra of the two reduced cofactors are identical, the combined emission from living tissues is often labelled NAD(P)H [6]. These characteristics have been utilised for the noninvasive interrogation of cellular metabolism since the 1950s[7]. Early experiments monitored NAD(P)H intensity using spectrofluorometry to address fundamental questions in respiratory chain activity and tissue oxygenation [8, 9]. The development of laser scanning confocal microscopy enabled subcellular imaging of NAD(P)H [10], providing insights into the role of mitochondrial dysfunction in disease [11]. These intensity‐based techniques continue to be applied on modern confocal and two‐photon microscopes [12, 13]. However, there is considerable interest in the extra dimensions of metabolic information that may be obtained from living systems through technologies that exploit the quantitative precision of time‐resolved NAD(P)H fluorescence [14, 15].

Following absorption of a photon, the average duration of excited state occupation defines the fluorescence lifetime of a molecule. This quantity is highly sensitive to its local environment and interactions, motivating the application of fluorescence lifetime imaging microscopy (FLIM) of NAD(P)H as a label‐free metabolic probe [6, 16]. In live cells, NAD(P)H FLIM typically resolves two lifetimes at each pixel: a short component ($\tau_{1}$ = 300–500 ps) associated with freely diffusing species, and a longer component ($\tau_{2}$ = 1500–4500 ps) attributed to enzyme‐bound forms [15, 17]. The relative abundance of the two species is also quantified, typically reported as the fraction of the emitting population exhibiting the longer lifetime ($\alpha_{2}$). Differences in these parameters have been described in a range of disease models over the last two decades [14], inspiring the development of novel approaches for clinical characterisation [18], monitoring [19] and diagnostics [20]. Despite these advances, NAD(P)H FLIM has yet to be established as a routine technique for detailed metabolic profiling as interpretation in terms of underlying cellular biochemical processes remains poorly understood. We address these issues here.

We have previously reported that the fluorescence lifetimes of NADH and NADPH in solution are differentially altered by the various possible configurations of binding to the oxidoreductases through which NAD and NADP impart their cellular functions [21]. The catalytic process of these enzymes involves conformational changes in both protein and cofactor [22, 23, 24]. In the open conformation of the enzyme, NAD(P) binds at its adenine end, leaving a mobile nicotinamide moiety; the region of the molecule responsible for its intrinsic fluorescence when reduced [25]. In the closed conformation, typically promoted by substrate binding, the nicotinamide becomes constrained in the active site for hydride transfer [26, 27]. Our previous studies revealed that the fluorescence lifetimes of bound NADH and NADPH are sensitive to these conformations [21]. The ~400 ps lifetime of free NADH increased to 1340(±40)ps in the open conformation and 3200(±200)ps in substrate‐free closed conformations. The increases for NADPH were larger, from ~400 ps to 1590(±50)ps and 4400(±200)ps, respectively. The addition of reduced substrates (e.g. lactate for lactate dehydrogenase, isocitrate for isocitrate dehydrogenase) further increased each lifetime, to a maximum of 5300 ps for NADPH in the closed configuration. No redox transfer can occur in these abortive ternary complexes as both cofactor and substrate are reduced. Catalytically productive ternary complexes with oxidised substrates were not, however, characterised at the time.

In this study, we have investigated the role that the above assortment of cofactor binding configurations plays in NAD(P)H FLIM measurements on living cells. Time‐resolved fluorescence anisotropy imaging (trFAIM), mathematical modelling of redox‐dependent binding equilibria and computational modelling of the decay fitting process were combined with FLIM measurements in a range of living cell models with well‐defined redox characteristics, including oocytes, astrocyte‐neuron cocultures and cancer stem cells. A deeper understanding of the biomolecular processes influencing the NAD(P)H fluorescence lifetime will pave the way towards meaningful interpretation of FLIM experiments in terms of underlying cell metabolism, as applied to both the investigation of biological mechanisms and diagnostic measurements in a clinical setting.

## Materials and methods

### HEK293 cell cultures

Frozen HEK293 stocks (RRID:CVCL_0045) were purchased from the American Type Culture Collection (ATCC) via LGC Standards (Teddington, UK) and were confirmed as bacteria, fungus and mycoplasma free. Authentication was performed by the vendor using short tandem repeat (STR) profiling. Cells were grown as monolayers in Dulbecco's Modified Eagle Medium (DMEM, Thermo Fisher Scientific, Dartford, UK) containing fetal bovine serum (10%), glucose (25 mm), sodium pyruvate (1 mm), GlutaMAX supplement (2 mm) and antibiotic‐antimycotic (providing 100 units·mL^−1^ penicillin, 100 μg·mL^−1^ of streptomycin and 0.25 μg·mL^−1^ of amphotericin B) within sterile 75‐cm^2^ culture flasks (Nunc EasYFlask, Thermo Fisher Scientific) in a 37 °C, 5% CO_2_ incubator. Cells were plated for imaging in glass‐bottomed 35‐mm dishes (FluoroDish, World Precision Instruments, Hitchin, UK) at a density of 300 000 per dish.

### Time‐resolved fluorescence anisotropy imaging

trFAIM measurements were performed on a multimodal time‐resolved fluorescence microscope. This combined an 80 MHz, near‐infrared, femtosecond excitation source (Insight X3, Spectra Physics, Crewe, UK), DCS‐120 laser scanning unit (Becker & Hickl, Berlin, Germany), inverted microscope (Axio Observer 7, Zeiss, Cambridge, UK) with high (1.4) numerical aperture objective (Plan‐Apochromat 63x/1.4 Oil M27, Zeiss, Cambridge, UK), two ultrafast hybrid detectors (HPM‐100‐07, Becker & Hickl, Berlin, Germany) and time‐correlated single photon counting (TCSPC) electronics (SPC‐180NX, Becker & Hickl, Berlin, Germany). NAD(P)H fluorescence was acquired for 2 min per field of view with 720‐nm excitation and 440(±40)nm emission filtering. On‐sample powers were kept below 10 mW. Counts were histogrammed at 14.6‐ps time intervals. A polarising beamsplitter cube allowed images of the orthogonally polarised fluorescence signals $I_{||}$ and $I_{⊥}$ to be obtained simultaneously in separate detectors. Mitochondrial regions of interest within each image were defined by thresholding in imagej (National Institutes of Health, Bethesa, MD, USA) based on their bright, punctate NAD(P)H fluorescence. Nuclear regions of interest were drawn by hand, identified as a single dim region within each cell. The remaining nonbackground pixels constituted cytosolic fluorescence.

### Oocyte culture

Procedures were approved by the University College London Animal Welfare and Ethical Review Board (UCL AWERB) and performed in accordance with personal licences granted by the UK Home Office and the Animal (Scientific Procedures) Act of 1986. Germinal vesicle (GV) stage oocytes were recovered from the ovaries of hormone‐primed 4–5‐week‐old (C57Bl6xCBA)F1 hybrid mice as described previously [28]. Oocytes were released into embryo‐tested M2 culture media (Merck Life Science, Dorset, UK) containing IBMX (200 μm) to maintain meiotic arrest. Oocytes were washed three times in M2 media, and repeated pipetting (using glass pipettes) was performed to remove cumulus cells when required. Imaging was performed in M2 media containing glucose (5.6 mM) with or without sodium pyruvate (0.2 mm) and sodium lactate (10 mm).

### Cortical cocultures

Procedures were approved by UCL AWERB and performed in accordance with personal licences granted by the UK Home Office and the Animal (Scientific Procedures) Act of 1986. E17 embryos were extracted from an adult female rat, sacrificed using cervical dislocation in accordance with Home Office regulations. Heads were stored in 4°C phosphate‐buffered saline (PBS). The brains were dissected, and the cortices were isolated, crushed and incubated in papain solution (Merck, 0.4 mg·mL^−1^ in PBS) at 37 °C for 20 min. Postincubation, the tissue was treated with DNAse (Merck, 0.4 mg·mL^−1^) and triturated to dissociate the cells with an 18G blunt filter needle and syringe. The homogenised solution was centrifuged at 500 ***g*** for 5 min, and the cell pellet was resuspended in neurobasal medium supplemented with GlutaMAX, B‐27 (Thermo Fisher) and penicillin–streptomycin. Cells were plated on poly‐l‐lysine‐treated coverslips in the wells of a six‐well plate and incubated in 5% CO_2_ at 37 °C. Medium changes were performed 48 h after plating and every 3–4 days thereafter. Imaging was performed in recording buffer containing NaCl (150 mm), KCl (4.25 mm), NaH_2_PO_4_ (1.25 mm), NaHCO_3_ (4 mm), CaCl_2_ (1.2 mm), HEPES (10 mm), glucose (10 mm) and MgCl_2_ (1.2 mm).

### Transformed mesenchymal stem cells

A previously published experimental model of stem cell oncogenesis [29] was gifted by Dr. Juan Manuel Funes (UCL Cancer Institute). This was established via the genetic alteration of human primary adult mesenchymal stem cells (MSCs) isolated from the bone marrow of a healthy donor. Cells labelled 3H expressed the catalytic subunit of human telomerase (hTERT) alongside the E6 and E7 human papillomavirus (HPV) oncogenes. 4H additionally expressed SV40 small T antigen, with further expression of an oncogenic allele of H‐Ras (V12) producing the 5H cells. Each of the three cell types was maintained in Advanced DMEM (Thermo Fisher Scientific) supplemented with fetal bovine serum (10%), GlutaMAX (2 mm), penicillin (100 U·mL^−1^) and streptomycin (100 μg·mL^−1^) as monolayers within sterile 75‐cm^2^ tissue culture flasks (Nunc EasYFlask, Thermo Fisher Scientific) in a 37 °C, 5% CO_2_ incubator. For imaging, cells were transferred to sterile 22 mm round coverslips in the wells of a six‐well plate (Nunc, Thermo Fisher Scientific), seeded at a density of 300 000 cells per well. Coverslips were held at the microscope using a custom made imaging chamber [30] with cells bathed in HEPES‐buffered, phenol red free DMEM (Thermo Fisher Scientific). Seven coverslips were imaged for each cell type, providing *n* = 22, 23 and 25 images for the 3H, 4H and 5H cells respectively.

### Metabolic assays

Oxygen consumption rates were measured by high‐resolution respirometry (Oxygraph‐2k, Oroboros Instruments, Innsbruck, Austria). Cells were collected by centrifugation and resuspended in Dulbecco's Modified Eagle Medium with HEPES (25 mm) replacing sodium bicarbonate as the buffer. Cell density was counted for normalisation using a haemocytometer. After measuring the baseline (routine) respiration rate, oligomycin A (2.5 μm), FCCP (2 μm) and antimycin A (2.5 μm) were sequentially added to obtain leak, maximally uncoupled and nonmitochondrial oxygen consumption rates, respectively [31, 32]. Glycolysis was assayed using the Seahorse XF Glycolysis Stress Test (Agilent, Harwell, UK) according to the manufacturer instructions. This quantified the extracellular acidification rate (ECAR) in glucose‐free conditions, following glucose stimulation and upon ATP synthase inhibition with oligomycin to allow determination of the glycolytic rate and total glycolytic capacity.

### Fluorescence lifetime imaging microscopy

Standard NAD(P)H FLIM was performed on an upright two‐photon microscope (Zeiss, Cambridge, UK) with a 1.0 NA 40× water‐dipping objective as previously described [17, 33, 34, 35, 36]. Excitation (<10 mW on‐sample) was provided by a Ti:sapphire laser (Chameleon Ultra II, Coherent, Cambridge, UK) tuned to 720 nm. Emission events were registered through a 460(±25) nm filter by an external detector (HPM‐100‐40, Becker & Hickl) attached to a TCSPC electronics module (SPC‐830, Becker & Hickl) with a histogram bin width of 48.8 ps. Scanning was performed continuously for 2 min with a pixel dwell time of 1.6 μs.

In oocytes, tetramethylrhodamine methyl ester (TMRM, 25 nm) was present to aid the identification of mitochondrial pixels. Its fluorescence was collected for a 10s burst using a 610(±30) nm emission filter with excitation provided at the same wavelength as NAD(P)H to avoid possible chromatic aberration. The 585(±15) nm emission spectrum of TMRM ensured its fluorescence did not contaminate the NAD(P)H images. As in the trFAIM experiments, nuclear regions were identified from their characteristically lower NAD(P)H fluorescence.

### Decay curve fitting

Standard pixel‐by‐pixel fitting of NAD(P)H FLIM data was performed in SPCImage 7.4 (Becker & Hickl, Berlin, Germany). All other decays were fit in MATLAB R2019a (The Mathworks, Cambridge, UK) using the lsqnonlin() function. In both cases, the decay model underwent periodic convolution with an instrument response function (IRF) measured using the second harmonic generation of a potassium dihydrogen phosphate (KDP) crystal at 920 nm, grown by leaving an aqueous molar solution on a coverslip to evaporate. The temporal position of the IRF was included as a freely varying parameter in each fit. Under the high numerical aperture excitation and collection conditions (NA/n~1) utilised here, the orthogonally polarised fluorescence decays (parallel and perpendicular to the polarisation of the excitation light) were described by [37, 38],

(1) $$I_{||}\left(t\right)=\frac{I\left(t\right)}{2}\left[1+R\left(t\right)\right]$$

(2) $$I_{⊥}\left(t\right)=\frac{I\left(t\right)}{2}\left[1-R\left(t\right)\right]$$

where $I\left(t\right)$ is the intensity decay resulting from loss of the excited state population, and $R\left(t\right)$ is the anisotropy decay resulting from diffusion‐induced depolarisation. The applicability of these functional forms, including the absence of polarisation dependent detection sensitivity (G=1) [39], was verified experimentally using measurements on NADH in solution (see Fig. S1). For trFAIM experiments, the two datasets were first summed to eliminate $R\left(t\right)$ and the fluorescence lifetimes $\tau_{i}$ and amplitudes $\alpha_{i}$ were extracted by fitting a multiexponential decay function,

(3) $$I\left(t\right)=Z+I\left(0\right)\sum_{i}\alpha_{i}\exp\left(-\frac{t}{\tau_{i}}\right)$$

where Z accounted for time‐uncorrelated background. The $I\left(t\right)$ parameters were then held constant when extracting the $R\left(t\right)$ parameters through simultaneous fitting of Eqs. 1 and 2 to the polarisation‐resolved decay data. This employed the associated anisotropy function.

(4) $$R\left(t\right)=\frac{\sum_{i}\alpha_{i}\exp\left(-\frac{t}{\tau_{i}}\right)R_{i}\left(0\right)\exp\left(-\frac{t}{\tau_{i}^{\mathrm{rot}}}\right)}{\sum_{i}\alpha_{i}\exp\left(-\frac{t}{\tau_{i}}\right)}$$

where $\tau_{i}^{\mathrm{rot}}$ are the rotational correlation times of each species. To account for local motion, the ‘wobbling in a cone’ model [21, 40, 41, 42] was later introduced.

(5) $$R\left(t\right)=\frac{\sum_{i}\alpha_{i}\exp\left(-\frac{t}{\tau_{i}}\right)R_{i}\left(0\right)\left[B_{i}\exp\left(-\frac{t}{\tau_{i}^{\text{local}}}\right)+\left\{1-B_{i}\right\}\exp\left(-\frac{t}{\tau_{\text{slow}}^{\mathrm{rot}}}\right)\right]}{\sum_{i}\alpha_{i}\exp\left(-\frac{t}{\tau_{i}}\right)}$$

### Synthetic decay curve generation

In MATLAB R2019a (The Mathworks, Cambridge, UK), multiexponential decay functions were generated (Eq. 3) with the amplitudes $\alpha_{i}$ provided by the solution to our steady‐state conformational equilibrium models at a given NAD(P)+/NAD(P)H ratio. The lifetimes $\tau_{i}$ were estimated from a combination of our live‐cell experiments and those obtained previously in solution (Table S1). The decay was convolved with the measured IRF of our FLIM system, scaled such that the peak bin had 500 counts, and a constant background of 10 counts was added; typical of our FLIM measurements. The distribution of expected fit parameters at each [NAD(P)+]:[NAD(P)H] was then determined by repeating, 1000 times, the addition of Poisson noise using the poissrnd() function followed by biexponential decay fitting.

### Statistical analysis

Fitting algorithms sought to minimise the reduced chi‐squared function.

(6) $$\chi_{R}^{2}=\frac{1}{m-l}\sum_{k=1}^{m}\frac{{\left[I_{\text{data}}\left(t_{k}\right)-I\left(t_{k}\right)\right]}^{2}}{I_{\text{data}}\left(t_{k}\right)}$$

where m is the total number of time bins k, and l is the number of free parameters in the model. We performed statistical tests to determine whether the introduction of additional model parameters was statistically justified, evaluating the *F* distribution at the ratio of $\chi_{R}^{2}$ values of the two models under comparison [43]. This process is illustrated in Fig. S2. Confidence intervals for each fitting parameter were calculated by varying them until $\chi_{R}^{2}$ increased to a critical value determined by the *F* statistic at *P* = 0.34, representing one standard deviation, while holding all others constant. Differences in FLIM parameter values between groups were assessed for statistical significance using two‐tailed Student's *t*‐tests.

## Results

### Enzyme‐bound species with fluorescence lifetimes both longer and shorter than free NAD(P)H are present in living cells

**Fig. 1:**
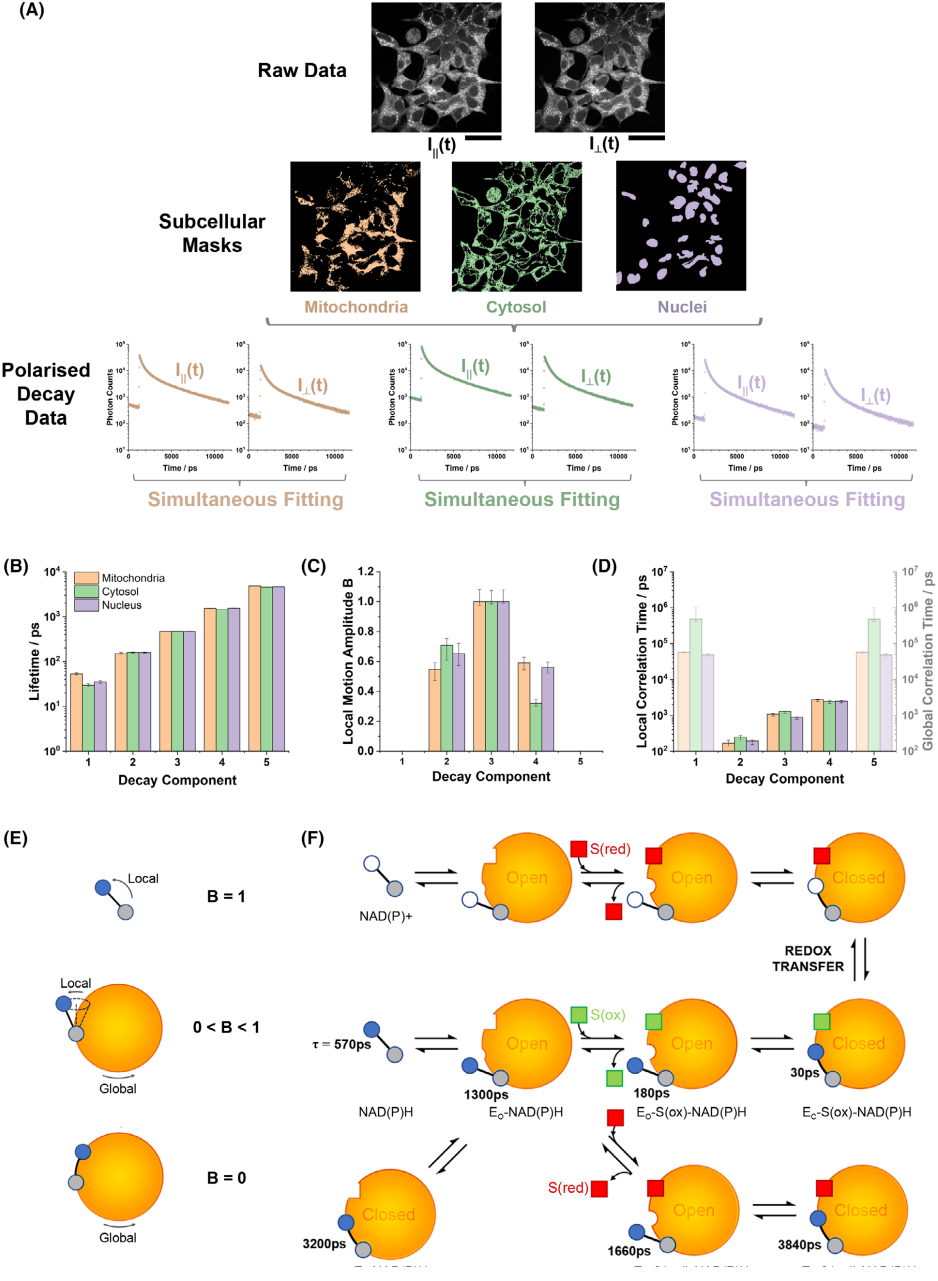
Time‐resolved fluorescence anisotropy imaging (trFAIM) of NAD(P)H in HEK293 cells. (A) Mitochondrial, cytosolic, and nuclear masks derived from their contrasting fluorescence intensities (scale bar 100 μm). Orthogonally polarised decay datasets were extracted by summing all photons in each image for each subcellular region. (B) The fluorescence lifetimes of the five resolved species and (C, D) their anisotropy decay parameters. Error bars indicate mean standard deviation confidence intervals (across *n* = 18 images from 6 dishes). (E) Schematic diagram indicating the relationship between the B parameter and the degree of local motion and (F) the generalised oxidoreductase reaction mechanism, including estimated fluorescence lifetimes of NADH in key binding configurations. Blue and white circles represent nicotinamide in the reduced and oxidised state, and grey circles the adenine moiety. Red and green squares represent the substrate of the enzyme in its reduced and oxidised form respectively.

We performed trFAIM to identify the range of NAD(P)H‐binding configurations present in living cells. This technique records a separate fluorescence decay for emission polarised parallel and perpendicular to that of the excitation light [44]. The application of appropriate fitting models then reveals both the fluorescence lifetime and rotational correlation time (inversely proportional to the rotational diffusion coefficient) for each species present in a heterogeneous population [21, 42]. As pixel‐by‐pixel fitting of NAD(P)H fluorescence decays in live cells can typically resolve only two decay components, we increased signal to noise by summing emission from mitochondrial, cytosolic and nuclear pixels into three separate decay curves per image, on average containing 5.0(±0.2) × 10^6^, 7.6(±0.3) × 10^6^ and 2.2(±0.1) × 10^6^ photon counts, respectively. These compartments were considered separately as their clearly contrasting NAD(P)H fluorescence intensities may imply distinct metabolic profiles. In contrast to the two lifetimes resolved with pixel‐by‐pixel fitting, where photon counts of the order of 10^3^ are typical [17], we were here able to resolve five decay components in HEK293 cells (Fig. 1; Table S2). Lifetimes were consistent across all the subcellular compartments: between 151–159 ps, 468–472 ps, 1441–1535 ps and 4579–4828 ps. Detection of a previously unobserved fast species with a 30–53 ps fluorescence lifetime was enabled by the fivefold improved time resolution of our trFAIM system compared to our standard FLIM apparatus [17, 33, 34, 35, 36].

We next quantified the rotational diffusion dynamics of each of the five identified species. We first considered a standard composite anisotropy model in which each species exhibited a single rotational correlation time (Table S3). Based on the Stokes–Einstein‐Debye equation [45], proteins that bind NAD(P)H (10^2^–10^4^ kDa [46]) should have rotational correlation times of 30 ns to 3 μs (assuming an aqueous environment and a typical 1.35 g cm^−3^ protein density [47]). The values corresponding to the species with lifetimes of 30–53 ps, 1441–1535 ps and 4579–4828 ps were generally consistent with this range, whereas the 151–159 ps and 468–472 ps species exhibited more rapid depolarisation. Based upon our previous studies in solution, this may reflect free diffusion of NAD(P)H in solution or the rapid local motion of NAD(P)H bound to an open conformation enzyme where the nicotinamide is unconstrained [21]. We therefore introduced the ‘wobbling in a cone’ model to quantify this behaviour [40, 41, 42], where a parameter B describes the level of rotational freedom (B=0 for no local motion, B= 1 for free rotation, 0<B<1 for local motion while constrained). Introducing this significantly improved fit quality ($\chi_{R}^{2}$ across all datasets reducing from 1.56 to 1.33, *F*‐test *P* < 10^−5^, *n* = 54). The B parameter was determined as 1 for the 468–472 ps lifetime component (Table S4). In contrast, *B* = 0.55–0.71 and 0.32–0.59 for the 151–159 ps and 1441–1535 ps components. Our prior knowledge allowed us to constrain the fit by fixing B at zero for the 4579–4828 ps component, given that such long lifetimes only resulted from fully bound cofactors in solution [21]. Surprisingly, B= 0 was also returned for the 30–53 ps species. The assignment of this, and the 151–159 ps species, as enzyme‐bound challenge the long‐held assumption that sub‐ns lifetimes in NAD(P)H FLIM purely reflect the free cofactor.

### Species in the enzyme‐bound population show differential responses to NAD(P) redox state

**Fig. 2:**
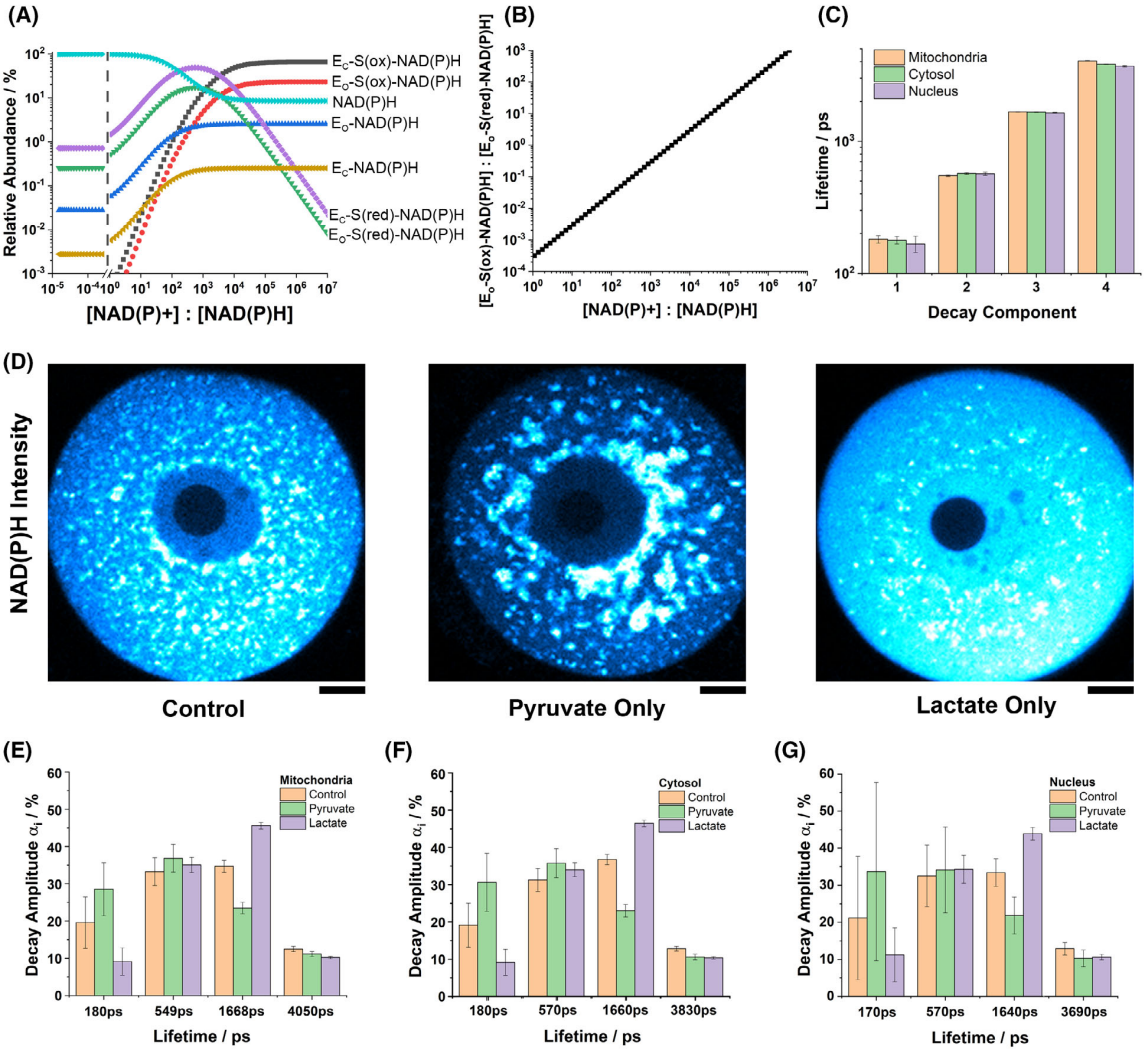
Redox‐controlled conformational and binding equilibria in mammalian oocytes. (A) Variation in the relative abundance of the binding configurations with changes in [NAD+]:[NADH] (or [NADP+]:[NADPH]), predicted by a generalised model of oxidoreductase catalysis. (B) The concentration ratio of the catalytic E_O_‐S(ox)‐NAD(P)H to abortive E_O_‐S(red)‐NAD(P)H complexes followed an approximate power law relationship with the redox state. (C) This prediction was tested in standard NAD(P)H FLIM measurements on oocytes (*n* = 17, 10 and 11 cells for control, pyruvate and lactate conditions respectively), where four fluorescence lifetimes could be resolved, ranging from 170 ps to 4050 ps. (D) NAD(P)H intensity images demonstrating the striking impact of media composition on the cellular NAD(P)H signal in oocytes (scale bar 10 μm), with lactate and pyruvate maximising and minimising the fluorescence, respectively. (E–G) Decay amplitudes in each subcellular region in response to changes in media composition (error bars represent standard deviation confidence intervals).

By interpreting the trFAIM results using our prior studies in solution [21], we could assign different binding configurations to the decay components observed here inside cells. The longest lifetime (4579–4828 ps) would correspond to closed, catalytically unproductive conformations with either a reduced substrate or no substrate, hereafter labelled E_C_‐S(red)‐NAD(P)H and E_C_‐NAD(P)H respectively. The 1441–1535 ps component represented open conformations, likely an average of binary and noncatalytic (reduced substrate) ternary complexes, E_O_‐NAD(P)H and E_O_‐S(red)‐NAD(P)H. Additional routes of excited state decay must be available in the shorter lifetime (30–53 ps and 151–159 ps) bound states. These were likely ternary complexes with oxidised substrates, where quenching by photoinduced electron transfer has previously been observed in fluorescence intensity measurements [48]. As this process is distance dependent [49], the longer of these would represent the open form, E_O_‐S(ox)‐NAD(P)H, and the shorter the closed form, E_C_‐S(ox)‐NAD(P)H, supported by the lack of local motion implied by the trFAIM data. We validated these configurational assignments by generating testable predictions through mathematical modelling of the binding and conformational equilibrium (Appendix S1). When parameterised using rate constants from in‐solution temperature jump spectroscopy experiments [26] (Table S5), our generalised model of oxidoreductase catalysis revealed dynamic variation in the steady‐state concentrations of each bound species with [NAD+]:[NADH] or [NADP+]:[NADPH] (Fig. 2). Notably, the model predicted an increase in the relative abundance of shorter lifetime E_O_‐S(ox)‐NAD(P)H and E_C_‐S(ox)‐NAD(P)H species and decrease of the longer lifetime E_O_‐S(red)‐NAD(P)H and E_C_‐S(red)‐NAD(P)H complexes as the oxidation of the cofactor pool increased. The ratio of the open forms of these species approximated to a straight line on a log–log plot against [NAD(P)+]:[NAD(P)H].

We tested the key prediction from our model on a dataset previously acquired from studies of mammalian oocytes obtained using our standard FLIM system [17, 33, 34, 35, 36, 50]. These highly specialised cells do not use glucose to produce ATP by glycolysis. Instead, exogeneous pyruvate or pyruvate derived from exogeneous lactate is imported into the mitochondria for further metabolism in the tricarboxylic acid (TCA) cycle [51]. This allows straightforward experimental control of [NAD+]:[NADH] through the choice of exogeneous substrate [52]. We compared FLIM measurements on oocytes incubated under control conditions (glucose, lactate and pyruvate) to those incubated in only lactate or only pyruvate, alongside glucose. Incubation with lactate resulted in exceptionally bright cytosolic fluorescence which became darker when the media was switched to pyruvate, reflecting the directionality of lactate dehydrogenase in each condition. Summing photon counts from all mitochondrial, cytosolic and nuclear pixels in each image initially revealed three decay components in each compartment (Table S6). Under control conditions, the shortest lifetime remained constant between compartments (~320 ps), with the two longer lifetimes varying slightly (1160–1270 ps, 3150–3530 ps). Altered substrate supply caused variations in all three lifetimes, suggesting that there were more than three underlying fluorescent species whose relative contributions were changing. To further resolve these, a global analysis model with lifetimes fixed between conditions, but amplitudes free to vary, was applied. This extracted four components (Table S7) and significantly improved fit quality ($\chi_{R}^{2}$ from 1.67 to 1.48, *F*‐test *P* < 10^−19^, *n* = 38). The shortest three lifetimes were consistent across compartments (170–180 ps, 550–570 ps, 1640–1670 ps), while the longest varied (4050 ps in mitochondria, 3830 ps in cytosol and 3690 ps in the nucleus). We were unable to identify the lifetimes of individual contributors to this longest species as no further decay components could be resolved; a five‐component global fit giving $\chi_{R}^{2}=$ 17.6. As in the trFAIM measurements in HEK293 cells, this likely reflected the lack of sensitivity of the experiment to longer decay times, particularly as only around 10% of the excited state population exhibited these longest lifetimes. In contrast to the trFAIM, no 30–53 ps component could be resolved due to the fivefold slower time resolution of our standard FLIM instrumentation. The relative abundance of the four lifetimes was similar in each subcellular compartment. In the control media, these were 19–21%, 31–33%, 33–37% and 12–13% (shortest to longest decay components). Switching between media containing pyruvate and lactate left the amplitudes of the 550–570 ps and 3690–4050 ps components relatively unchanged. However, those of the other two species changed dramatically, and in opposing directions. In pyruvate‐only media, the amplitude of the 170–180 ps lifetime species increased to 29–34% and that of the 1640–1668 ps species decreased to 22–23%. In lactate‐only media, these instead decreased to 9–11% and increased to 44–46% respectively. This recreated the predictions of our model, providing support for the configurational assignment we outlined above, with the 170–180 ps and 1640–1668 ps decay components reflecting the E_O_‐S(ox)‐NAD(P)H and E_O_‐S(red)‐NAD(P)H species, respectively.

### The redox‐driven binding and conformational equilibrium controls the biexponential decay parameters output by NAD(P)H FLIM

**Fig. 3:**
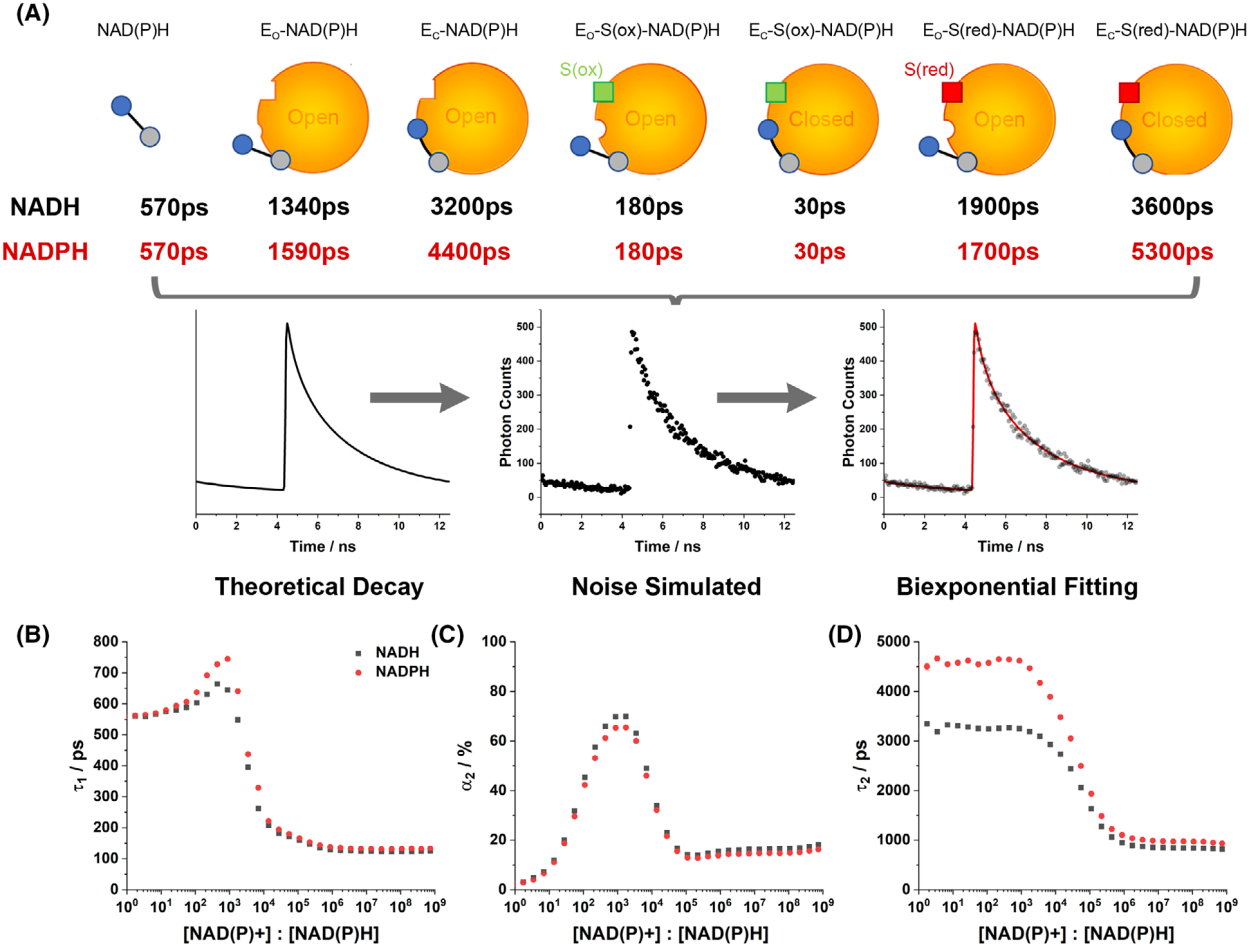
Simulating the conditions of pixel‐by‐pixel NAD(P)H FLIM. (A) Schematic diagrams of the generation of fluorescence decay curves based on the model‐predicted values for the relative abundances of each NAD(P)H binding configuration. As in Fig. 1, blue circles represent reduced nicotinamide and grey circles the adenine moiety. Red and green squares represent the substrate of the enzyme in its reduced and oxidised form. Lifetimes for each configuration were estimated for both NADH and NADPH from the results of the present study and those obtained previously in solution [21]. 1000 iterations of noise generation and fitting were performed at varying redox ratios using the results of the conformational equilibrium modelling (Fig. 2A), allowing the canonical biexponential FLIM parameters (B) $\tau_{1}$, (C) $\alpha_{2}$ and (D) $\tau_{2}$ to be plotted.

We next performed computational simulations of the FLIM fitting process to understand how the heterogeneous mix of binding configurations would be reflected in the simplified set of parameters ($\tau_{1}$, $\tau_{2}$ and $\alpha_{2}$) typically reported by NAD(P)H FLIM. As expected, fluorescence decays simulated with seven decay components but signal‐to‐noise levels reflecting pixel‐by‐pixel fitting were adequately described by just two components (Fig. 3). With underlying lifetimes reflecting those we predicted for NADH (Table S1), the shorter component ($\tau_{1}$) exhibited the lifetime of free NADH only at very low [NAD+]:[NADH], instead reflecting that of E_O_‐S(ox)‐NAD(P)H (180 ps) at high ratios. It peaked between these extremes at ~720 ps due to contributions from E_O_‐NAD(P)H and E_O_‐S(red)‐NAD(P)H, meaning $\alpha_{2}$ would no longer accurately report the proportion of enzyme‐bound NADH. The longer lifetime ($\tau_{2}$) ranged from 3500 ps at low [NAD+]:[NADH] to 850 ps at high ratios. The longer of these reflected the combined contribution of E_O_‐S(red)‐NAD(P)H and E_C_‐S(red)‐NAD(P)H, while the shorter value likely resulted from a mixture of E_O_‐NAD(P)H and free NADH no longer assigned to $\tau_{1}$. With the underlying lifetimes switched to reflect those we predicted for NADPH, the behaviour of $\tau_{1}$ and $\alpha_{2}$ with [NADP+]:[NADPH] was broadly unchanged. $\tau_{2}$ was similar as for NADH at more oxidised redox states, but it reached a much larger value of around 4500 ps as the cofactor pool became more reduced, due to the intrinsically longer lifetimes of many of the NADPH‐associated bound species that we previously observed in solution [21]. These results demonstrated that, while the biexponential analysis typically applied to NAD(P)H FLIM data could not separately resolve the heterogeneous population of bound species underlying it, correlations with the NAD and NADP redox states nevertheless existed.

We performed measurements on a range of multicellular systems with predictable differences in redox state to experimentally test these relationships. In oocytes with their surrounding cumulus cells still present, $\tau_{1}$ was significantly higher in the oocytes, at 650(±20)ps compared to 470(±20)ps (Fig. S3). Our modelling suggested that such large values of $\tau_{1}$ in the oocyte must imply a lower [NAD(P)+]:[NAD(P)H] ratio. This was in agreement with the known contrasting directionality of lactate dehydrogenase in the two cell types, with cumulus cells secreting lactate (consuming NADH) and oocytes consuming it (producing NADH) [53]. Next, in co‐cultures of cortical neurons and astrocytes, we observed a longer $\tau_{2}$ in astrocytes compared to neurons (Fig. S4). Our modelling associated this with a lower [NAD+]:[NADH] in the astrocytes, a conclusion supported by equivalent measurements made with genetically encoded NAD sensors [54]. Lastly, in a mesenchymal stem cell model of oncogenesis [29], $\tau_{1}$, $\alpha_{2}$ and $\tau_{2}$ were identical in cell lines that expressed four or five oncogenes (denoted 4H and 5H), but all were significantly lower in cells expressing only three (3H, see Fig. S5). These differences mirrored the oxygen consumption rates of the three cell lines (Table S8), with 3H respiring more slowly than 4H and 5H. As rates of respiration and redox state are closely linked [55], this reinforced the importance of cellular redox state in controlling the fluorescence decay of NAD(P)H. However, this result also highlighted the limitations of applying a single redox equilibrium model (NAD or NADP alone) to interpret intracellular measurements involving both. Our model predicted that $\tau_{2}$ should decrease with the increased [NAD+]:[NADH] expected in cells with higher respirations rates. Observation of the opposite likely reflected the greater contribution of bound NADPH, with its longer lifetime [17], to the combined NAD(P)H signal upon NAD oxidation. Future work must therefore model the interplay of the NAD and NADP pools, their redox states and fluorescence characteristics, but this is beyond the scope of the present study.

## Discussion

By combining trFAIM with FLIM measurements in a range of living cell models under different conditions, mathematical modelling of redox‐dependent binding equilibria, and computational modelling of the decay fitting process, we have found that the cellular [NAD+]:[NADH] and [NADP+]:[NADPH] redox balances control steady‐state equilibria of binding configurations for each cofactor, dictating the lifetime distribution of fluorescent species present. This results in a fluorescence decay with at least seven components, which approximates to a biexponential under the limited signal‐to‐noise conditions when imaging living samples. We then showed that the lifetimes of the two components ($\tau_{1}$ and $\tau_{2}$) and their relative weighting ($\alpha_{2}$) are each sensitive to the redox states of the NAD or NADP pools.

Our work highlights the additional level of molecular scale detail available by introducing polarisation resolution into metabolic FLIM measurements. Time‐resolved fluorescence anisotropy has been applied sporadically to NAD(P)H in living cells over the last two decades [56]. Most notably, Vishwasrao et al. used it in 2005 to observe alterations in the rate of fluorescence depolarisation in normoxic and hypoxic brain [57]. A decreased mean NAD(P)H rotational correlation time upon hypoxia was interpreted as a decrease in the bulk viscosity of the tissue, attributed to osmotic swelling. This can be interpreted differently considering our results. By reducing the supply of oxygen to the electron transport chain, hypoxia would cause a decrease in [NAD+]:[NADH], driving the binding equilibrium towards the more rapidly rotating free NAD(P)H. Furthermore, bulk viscosity is unlikely to be representative of the immediate local viscosity of the fluorescent nicotinamide moiety [45]. The cellular environment is far from homogeneous at these length scales, and the nicotinamide in free NAD(P)H or partially bound in an open conformation enzyme is likely to exist in an aqueous nanodomain [58]. In contrast, while the enzymes to which NAD(P)H binds are large enough to be affected by the bulk viscosity, polarised TCSPC is relatively insensitive to these long rotational correlation times. This may explain the lack of impact of the contrasting bulk viscosities of mitochondria, cytosol, and nucleus on our fluorescence anisotropy measurements [59]. A more advanced approach to segmentation, perhaps utilising extrinsic stains or accounting for border pixels, may increase sensitivity in this regard.

The two decay components typically resolved in NAD(P)H FLIM experiments have been widely attributed to free and enzyme‐bound NAD(P)H [6, 14, 15], but our study suggests this to be an oversimplification. Our results implied the presence of enzyme‐bound forms with shorter lifetimes than free NAD(P)H, specifically ternary complexes with oxidised substrates that likely accelerate non‐radiative decay through photoinduced electron transfer [48]. One form had a very short lifetime (~30–53 ps) that may fall below the time resolution of many standard FLIM systems, while another (151–159 ps) likely contributes to the faster decay component commonly assigned to free NAD(P)H. We demonstrated that the range of NAD(P)H binding configurations that contribute to its emission in living cells each exhibit distinct fluorescence lifetimes. These configurations correspond to different conformations in the catalytic action of oxidoreductase enzymes, and their relative steady‐state abundances are determined by the [NAD+]:[NADH] and [NADP+]:[NADPH] redox balances. This implies that the well‐established correlations between NAD(P)H FLIM measurements and metabolism [6, 14, 15] are underpinned by metabolically induced alterations in these ratios.

A mix of NADH and NADPH contributes to the observed signal in live cell NAD(P)H FLIM, where we previously observed their enzyme‐bound forms to be represented by lifetimes of ~1500 ps and ~ 4400 ps respectively, and the value of $\tau_{2}$ in a biexponential fit reflecting the relative proportion of these two species [17]. Our results here serve to provide a mechanism for this. Our modelling implied that the long lifetime non‐catalytic E_O_‐S(red)‐NAD(P)H and E_C_‐S(red)‐NAD(P)H species dominate when the cofactor pool is highly reduced. Such conditions are known to be maintained for the NADP pool to fulfil its role in supporting reductive biosynthesis [60]. The limiting lifetime value at low [NADP+]:[NADPH] was ~4500 ps, in striking agreement with our previous predictions for the intracellular lifetime of bound NADPH [17]. Conversely, the NAD pool is maintained in a more oxidised state to provide electron acceptors for catabolic redox reactions [54]. However, the limiting lifetime value at high [NAD+]:[NADH] here was ~970 ps. This was significantly shorter than our previous prediction for the fluorescence lifetime of bound NADH in cells, which was closely reflected by the 1340 ps lifetime of the E_O_‐NAD(P)H species. This shortening most probably resulted from an overestimation of the population of species with shorter lifetimes (catalytic ternary complexes and free NADH) under the most oxidised conditions. Furthermore, this limiting lifetime was reached at much higher [NAD+]:[NADH] ratios (>10^7^) than expected (~10^2^) inside the cell [54]. These disagreements likely resulted from our choice of rate constants. In the absence of more appropriate data, it was necessary to assume that the binding and conformational kinetics of the enzyme in our model were invariant to the redox states of the bound cofactor or substrate. The values were also estimated from the only detailed characterisation available, performed on a lactate dehydrogenase isoform that favours the production of NADH (and pyruvate) [26]. Enzymes with rate constants that favour NAD+ production via preferential binding of NADH and oxidised substrate must instead dominate inside the cell to maintain the NAD pool in an oxidised state.

The model we developed here successfully fulfilled its initial purpose of aiding the assignment of each fluorescent NAD(P)H species to the various oxidoreductase binding configurations. A more general model based upon the individual redox states of the NAD and NADP pools, possibly allowing them to be deduced from an NAD(P)H fluorescence decay measurement, would require representation of the diverse range of NADH and NADPH‐associated enzymes present in the cell [61]. Although not all oxidoreductases involve the reversible binding of a substrate, the generalised reaction mechanism upon which we based our model could still be utilised through an appropriate choice of rate constants. For example, in complex I of the respiratory chain, the redox partner of NADH is a prosthetic flavin group, so the rate of substrate unbinding would be zero. A similar approach could account for the existence of oxidoreductases that do not utilise the open‐closed conformational transition. These are, however, hypothetical, as this is widely observed across oxidoreductases [62, 63, 64, 65, 66, 67], including complex I [68].

Parameterising a model linking NAD(P)H FLIM to the cellular NAD and NADP redox states would require quantifying the conformational kinetics of a range of enzymes beyond those thus far performed on lactate dehydrogenase alone [26]. Precision characterisation of the corresponding bound NAD(P)H decay kinetics may require faster techniques than we applied here, such as transient absorption [69] or TCSPC with ultrafast detectors [70], given our observation of sub‐100 ps lifetimes. This also raises the question of how such a large array of different enzymes could be analytically represented. Yang et al. have made advances in this direction [71], developing a simplified model that allowed flux through the mitochondrial respiratory chain to be predicted based on canonical biexponential NAD(P)H FLIM readouts. Their coarse graining procedure allocated all oxidoreductases within the cell into two categories; those that reduced NAD(P) + to NAD(P)H, and those that oxidised NAD(P)H to NAD(P)+. With the mechanistic insights from our present study hitherto unavailable to them, an ansatz assumption was made that these possess two distinct fluorescence lifetimes. Our own numerical analysis of their model (Appendix S2 and Fig. S6) revealed an inherent prediction that the NAD(P)H producing enzymes possess a longer fluorescence lifetime than the NAD(P)H consuming enzymes. Our results here provide evidence to support this, where we have shown that environments with high [NAD(P)+]:[NAD(P)H] ratios, which would require a higher abundance of the NAD(P)H consumers, do indeed exhibit a shorter value of $\tau_{2}$ in a biexponential NAD(P)H FLIM experiment, and vice versa. Integrating their innovative modelling approach with the detailed molecular scale insights we provide here may be a promising avenue of future research.

In conclusion, we have shown that NAD(P)H FLIM measurements respond to metabolism via sensitivity to metabolically induced alterations in the redox states of the NAD and NADP pools. These drive the equilibrium of an array of contrasting NAD(P)H binding configurations, each contributing distinct decay kinetics to a highly heterogeneous fluorescent population. This new knowledge can now facilitate the development of accurate metabolic models, both qualitative and quantitative, for interpreting NAD(P)H FLIM.

## Author contributions

NP prepared primary astrocyte‐neuron cocultures. TSB, NM, NP, ERW and MDES performed experiments. TSB, ERW and MDES analysed data. TSB, JC, GS, AJB and MRD supervised the work. All authors drafted the manuscript.

## Supporting information


**Appendix S1.** Model details.
**Appendix S2.** Comparisons with an existing coarse‐grained model of NAD(P)H FLIM.
**Fig. S1.** Polarised intensity decay measurements on a 1 mM solution of NADH in phosphate‐buffered saline.
**Fig. S2.** An example of the least‐squares fitting process.
**Fig. S3.** NAD(P)H FLIM of mammalian oocytes with intact cumulus cells.
**Fig. S4.** NAD(P)H FLIM of mixed cocultures of cortical neurons and astrocytes.
**Fig. S5.** NAD(P)H FLIM of a mesenchymal stem cell model of oncogenesis.
**Fig. S6.** Modelling experimentally determined parameter values.
**Table S1.** Lifetimes used in the simulation of pixel‐by‐pixel NAD(P)H fluorescence decays and their origin.
**Table S2.** Mean NAD(P)H fluorescence lifetimes in live HEK293 cells.
**Table S3.** Mean‐associated anisotropy decay fit parameters for NAD(P)H in HEK293 cells.
**Table S4.** Mean‐associated ‘wobbling in a cone’ anisotropy decay fit parameters for NAD(P)H in HEK293 cells.
**Table S5.** Values of parameters used to solve the redox equilibrium model.
**Table S6.** Mean NAD(P)H fluorescence decay parameters in subcellular compartments of mammalian oocytes.
**Table S7.** Mean NAD(P)H fluorescence decay parameters in mammalian oocytes with compartmentalised lifetimes shared between conditions.
**Table S8.** Metabolic characterisation of transformed mesenchymal stem cells.

## Data Availability

The data that support the findings of this study are available from the corresponding author (t.blacker@ucl.ac.uk) upon reasonable request.
