# Supplementary material for: Redox‐dependent binding and conformational equilibria govern the fluorescence decay of NAD(P)H in living cells
Source: FEBS Lett. 2025 Jul 25;599(19):2802–16. doi: 10.1002/1873-3468.70125 (PMC12519055; doi:10.1002/1873-3468.70125)
Supplement: Supplementary file 1 — Appendix S1. Model details. Appendix S2. Comparisons with an existing coarse‐grained model of NAD(P)H FLIM. Fig. S1. Polarised intensity decay measurements on a 1 mM solution of NADH in phosphate‐buffered saline. Fig. S2. An example of the least‐squares fitting process. Fig. S3. NAD(P)H FLIM of mammalian oocytes with intact cumulus cells. Fig. S4. NAD(P)H FLIM of mixed cocultures of cortical neurons and astrocytes. Fig. S5. NAD(P)H FLIM of a mesenchymal stem cell model of oncogenesis. Fig. S6. Modelling experimentally determined parameter values. Table S1. Lifetimes used in the simulation of pixel‐by‐pixel NAD(P)H fluorescence decays and their origin. Table S2. Mean NAD(P)H fluorescence lifetimes in live HEK293 cells. Table S3. Mean‐associated anisotropy decay fit parameters for NAD(P)H in HEK293 cells. Table S4. Mean‐associated ‘wobbling in a cone’ anisotropy decay fit parameters for NAD(P)H in HEK293 cells. Table S5. Values of parameters used to solve the redox equilibrium model. Table S6. Mean NAD(P)H fluorescence decay parameters in subcellular compartments of mammalian oocytes. Table S7. Mean NAD(P)H fluorescence decay parameters in mammalian oocytes with compartmentalised lifetimes shared between conditions. Table S8. Metabolic characterisation of transformed mesenchymal stem cells. [file FEB2-599-2802-s001.docx]

***Supporting Information***

**Redox-dependent binding and conformational equilibria govern the fluorescence decay of NAD(P)H in living cells**

Thomas S. Blacker^1,*^, Nimit Mistry^1^, Nicoletta Plotegher^2^, Elizabeth R. Westbrook^2^, Michael D. E. Sewell^2^, John Carroll^2^, Gyorgy Szabadkai^2^, Angus J. Bain^3^, Michael R. Duchen^2^

^1^Research Department of Structural & Molecular Biology, University College London, Gower Street, London WC1E 6BT, United Kingdom

^2^Research Department of Cell & Developmental Biology, University College London, Gower Street, London WC1E 6BT, United Kingdom

^3^Department of Physics & Astronomy, University College London, Gower Street, London WC1E 6BT, United Kingdom

^*^Corresponding Author: t.blacker@ucl.ac.uk


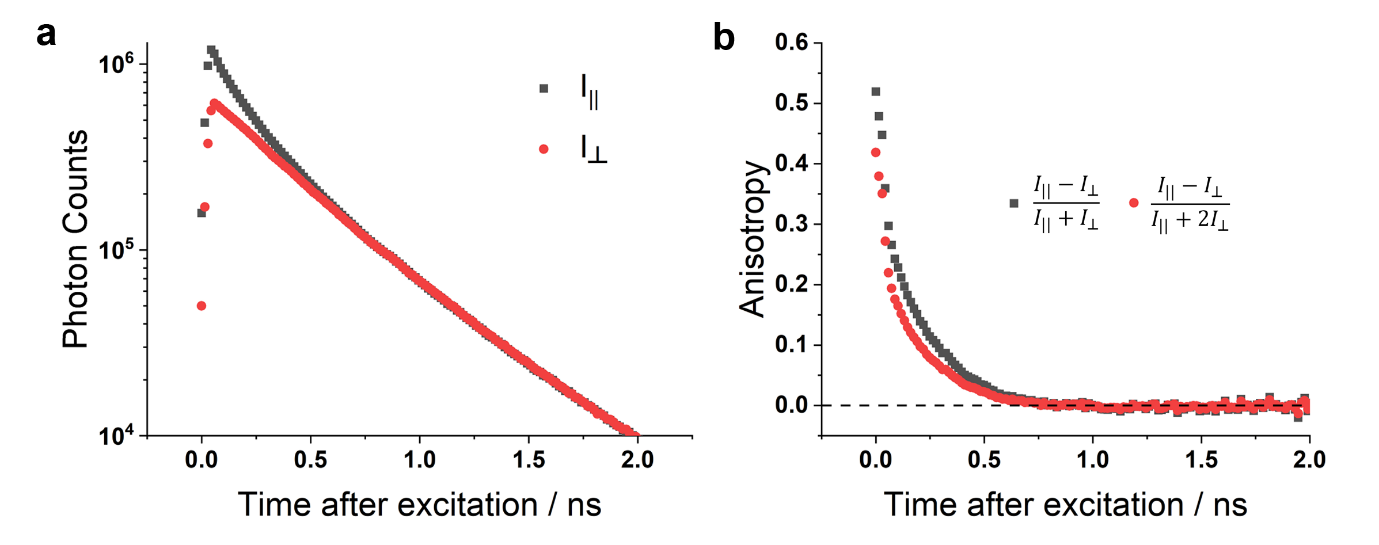


**Figure S1:** Polarised intensity decay measurements on a 1mM solution of NADH in phosphate buffered saline. (a) I_||_ and I_┴_ become equal as the time after excitation increases, indicating the absence of preferential detection for either polarisation (a “G factor” of unity). (b) Appropriately formulating the intensity term of the fluorescence anisotropy for the high numerical aperture conditions of our system[1,2] leads to the expected value[3] (approximately 0.52) of the initial two-photon anisotropy for NADH in aqueous solution (black data). Ignoring these effects by assuming plane polarised excitation causes an underestimation of the anisotropy (red data).

**Table S1:** Lifetimes used in the simulation of pixel-by-pixel NAD(P)H fluorescence decays and their origin

| **Species** | | **NADH lifetime / ps** | | **NADPH lifetime / ps** | | | **Origin** |
| --- | --- | --- | --- | --- | --- | --- | --- |
| NAD(P)H | | 570 | | 570 | | | Oocyte FLIM |
| E_O_-NAD(P)H | | 1340 | | 1590 | | | In-solution[4] |
| E_c_-NAD(P)H | | 3200 | | 4400 | | | In-solution[4] |
| E_O_-S(ox)-NAD(P)H | | 180 | | 180 | | | Oocyte FLIM |
| E_c_-S(ox)-NAD(P)H | | 30 | | 30 | | | HEK293 trFAIM |
| E_O_-S(red)-NAD(P)H | | 1900 | | 1700 | | | In-solution[4] |
| E_C_-S(red)-NAD(P)H | | 3600 | | 5300 | | | In-solution[4] |
| **Key** | | | | | | | |
| E | O | | C | | S(ox) | S(red) | |
| Enzyme | Open | | Closed | | Oxidised substrate | Reduced substrate | |


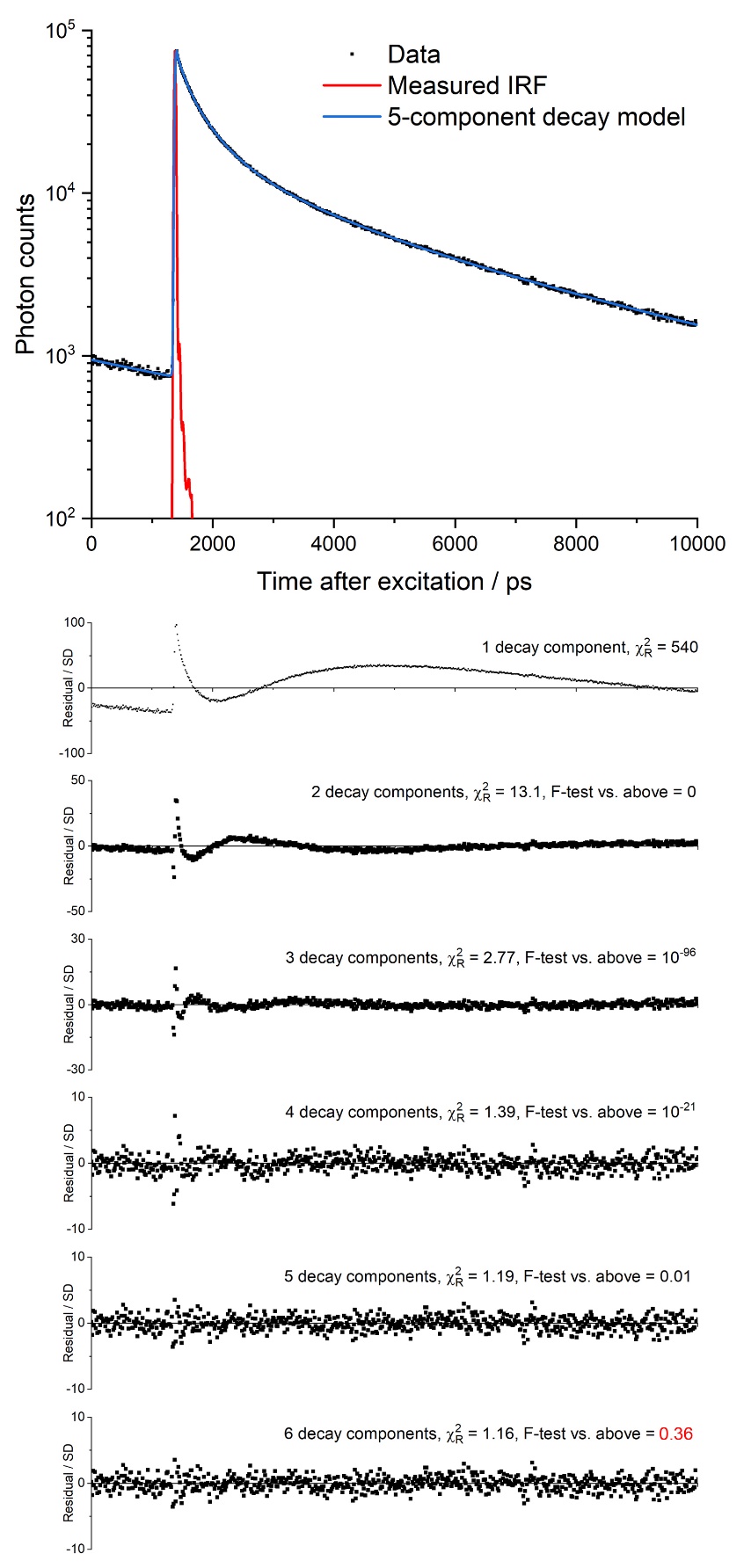


**Figure S2:** An example of the least-squares fitting process, here for cytosolic intensity decay data. Components were added to the model to improve the agreement between model and data, reflected by a decrease in the $\chi_{R}^{2}$ statistic. An F-test P value < 0.05 demonstrated that the magnitude of $\chi_{R}^{2}$ decrease warranted the application of the more complex model. The accepted model was that which minimised $\chi_{R}^{2}$ with a statistically justified level of complexity.

**Table S2:** Mean NAD(P)H fluorescence lifetimes in live HEK293 cells (n=18 images across 6 dishes) determined using trFAIM. Values in square brackets indicate mean standard deviation confidence intervals.

|  | **Mitochondria** | | **Cytosol** | | **Nucleus** | |
| --- | --- | --- | --- | --- | --- | --- |
| $\tau_{1}$ / ps | 53 | [51, 57] | 30 | [28, 32] | 35 | [33, 38] |
| $\tau_{2}$ / ps | 151 | [147, 160] | 159 | [154, 161] | 158 | [154, 161] |
| $\tau_{3}$ / ps | 468 | [465, 472] | 472 | [469, 475] | 470 | [467, 473] |
| $\tau_{4}$ / ps | 1520 | [1511, 1528] | 1441 | [1432, 1449] | 1535 | [1523, 1546] |
| $\tau_{5}$ / ps | 4828 | [4814, 4842] | 4579 | [4568, 4591] | 4652 | [4629, 4674] |
| $a_{1}$ / % | 21.7 | [20.6, 22.7] | 21.6 | [20.7, 22.5] | 21.8 | [20.5, 22.9] |
| $a_{2}$ / % | 22.9 | [22.4, 23.4] | 23.9 | [23.5, 24.2] | 27.9 | [27.3, 28.4] |
| $a_{3}$ / % | 30.9 | [30.7, 31.2] | 31.3 | [31.1, 31.5] | 33.7 | [33.5, 34.0] |
| $a_{4}$ / % | 14.0 | [13.9, 14.1] | 13.0 | [12.9, 13.1] | 10.7 | [10.6, 10.8] |
| $a_{5}$ / % | 10.5 | [10.4, 10.6] | 10.2 | [10.1, 10.3] | 6.0 | [5.9, 6.1] |
| $\chi_{R}^{2}$ | 1.57 | | 1.92 | | 1.29 | |

**Table S3:** Mean associated anisotropy decay fit parameters for NAD(P)H in HEK293 cells (n=18 images across 6 dishes). Values in square brackets indicate mean standard deviation confidence intervals.

|  | **Mitochondria** | | **Cytosol** | | **Nucleus** | |
| --- | --- | --- | --- | --- | --- | --- |
| $\tau_{1}^{\text{rot}}$ / ps | 93162 | [10681, 1e12] | 114696 | [42409, 1e12] | 188632 | [9064, 1e13] |
| $\tau_{2}^{\text{rot}}$ / ps | 417 | [233, 432] | 135 | [131, 170] | 315 | [250, 404] |
| $\tau_{3}^{\text{rot}}$ / ps | 908 | [846, 1076] | 624 | [584, 657] | 1197 | [1078, 1435] |
| $\tau_{4}^{\text{rot}}$ / ps | 236920 | [5872, 237543] | 4627 | [4457, 5121] | 86873 | [3610, 87274] |
| $\tau_{5}^{\text{rot}}$ / ps | 3059504 | [259248, 4e17] | 323112 | [75647, 324809] | 217757 | [57466, 224426] |
| $R_{1}^{0}$ | 0.51 | [0.37, 0.71] | 0.54 | [0.49, 0.56] | 0.53 | [0.17, 0.56] |
| $R_{2}^{0}$ | 0.46 | [0.43, 0.48] | 0.43 | [0.40, 0.45] | 0.49 | [0.46, 0.51] |
| $R_{3}^{0}$ | 0.35 | [0.34, 0.36] | 0.40 | [0.39, 0.41] | 0.34 | [0.33, 0.35] |
| $R_{4}^{0}$ | 0.49 | [0.48, 0.50] | 0.51 | [0.50, 0.52] | 0.47 | [0.46, 0.48] |
| $R_{5}^{0}$ | 0.45 | [0.44, 0.46] | 0.49 | [0.48, 0.50] | 0.49 | [0.48, 0.50] |
| $\chi_{R}^{2}$ | 1.37 | | 2.09 | | 1.23 | |

**Table S4:** Mean associated “wobbling in a cone” anisotropy decay fit parameters for NAD(P)H in HEK293 cells (n=18 images across 6 dishes). Values in square brackets indicate mean standard deviation confidence intervals.

|  | **Mitochondria** | | **Cytosol** | | **Nucleus** | |
| --- | --- | --- | --- | --- | --- | --- |
| $\tau_{1}^{\text{local}}$ / ps | ${(B}_{1}=0)$ | | ${(B}_{1}=0)$ | | ${(B}_{1}=0)$ | |
| $\tau_{2}^{\text{local}}$ / ps | 169 | [140, 203] | 246 | [210, 274] | 195 | [151, 209] |
| $\tau_{3}^{\text{local}}$ / ps | 1082 | [957, 1132] | 1291 | [1140, 1316] | 842 | [786, 906] |
| $\tau_{4}^{\text{local}}$ / ps | 2752 | [2449, 2821] | 2425 | [2177, 2618] | 2504 | [2230, 2649] |
| $\tau_{5}^{\text{slow}}$ / ps | 56939 | [52749, 58293] | 477725 | [416238, 1016075] | 48484 | [44824, 51323] |
| $R_{1}^{0}$ | 0.49 | [0.20, 0.51] | 0.54 | [0.50, 0.56] | 0.55 | [0.33, 0.57] |
| $R_{2}^{0}$ | 0.51 | [0.47, 0.52] | 0.50 | [0.48, 0.51] | 0.50 | [0.47, 0.52] |
| $R_{3}^{0}$ | 0.28 | [0.27, 0.29] | 0.29 | [0.28, 0.30] | 0.29 | [0.28, 0.30] |
| $R_{4}^{0}$ | 0.52 | [0.51, 0.53] | 0.51 | [0.50, 0.52] | 0.51 | [0.50, 0.52] |
| $R_{5}^{0}$ | 0.47 | [0.46, 0.48] | 0.42 | [0.41, 0.43] | 0.47 | [0.46, 0.48] |
| $B_{1}$ | 0 | [0, 0] | 0 | [0, 0] | 0 | [0, 0] |
| $B_{2}$ | 0.55 | [0.47, 0.49] | 0.71 | [0.61, 0.75] | 0.65 | [0.57, 0.72] |
| $B_{3}$ | 1 | [0.97, 1.1] | 1 | [0.99, 1.1] | 1 | [0.99, 1.13] |
| $B_{4}$ | 0.59 | [0.54, 0.63] | 0.32 | [0.30, 0.34] | 0.56 | [0.52, 0.60] |
| $B_{5}$ | 0 | [fixed] | 0 | [fixed] | 0 | [fixed] |
| $\chi_{R}^{2}$ | 1.33 | | 1.50 | | 1.10 | |

**Appendix S1:** Model Details

We consider a generalised oxidoreductase reaction mechanism in which redox transfer occurs in the closed enzyme conformation with both cofactor and substrate present. Both cofactor and substrate can only bind to an open enzyme, and closure is promoted by the binding of the substrate itself. This leads to the following scheme:

$$\begin{matrix} \\ \text{A} \\ \end{matrix}\begin{matrix} k_{1} \\ \rightleftharpoons\\ k_{-1} \end{matrix}\begin{matrix} \\ \text{A∙Eo} \\ \end{matrix}\begin{matrix} k_{2} \\ \rightleftharpoons\\ k_{-2} \end{matrix}\begin{matrix} \\ \text{A∙Eo∙S(red)} \\ \end{matrix}\begin{matrix} k_{3} \\ \rightleftharpoons\\ k_{-3} \end{matrix}\begin{matrix} \\ \text{A∙Ec∙S(red)} \\ \end{matrix}\begin{matrix} k_{4} \\ \rightleftharpoons\\ k_{-4} \end{matrix}\begin{matrix} \\ \text{B∙Ec∙S(ox)} \\ \end{matrix}\begin{matrix} k_{5} \\ \rightleftharpoons\\ k_{-5} \end{matrix}\begin{matrix} \\ \text{B∙Eo∙S(ox)} \\ \end{matrix}\begin{matrix} k_{6} \\ \rightleftharpoons\\ k_{-6} \end{matrix}\begin{matrix} \\ \text{B∙Eo} \\ \end{matrix}\begin{matrix} k_{7} \\ \rightleftharpoons\\ k_{-7} \end{matrix}\begin{matrix} \\ \text{B} \\ \end{matrix}$$

where $\text{A}$ is (oxidised) NAD(P)+, $\text{B}$ is (reduced) NAD(P)H, $\text{S}$(red) is the reduced substrate (e.g. lactate in the case of lactate dehydrogenase), $\text{S}$(ox) is the oxidised product (e.g. pyruvate) and $\text{o}$ and $\text{c}$ represent the open and closed conformations of the enzyme $\text{E}$. We also included the possibility of “abortive” binding configurations that are not catalytically productive:

$$\begin{matrix} \\ \text{A∙Eo} \\ \end{matrix}\begin{matrix} k_{i} \\ \rightleftharpoons\\ k_{-i} \end{matrix}\begin{matrix} \\ \text{A∙Eo∙S(ox)} \\ \end{matrix}\begin{matrix} k_{j} \\ \rightleftharpoons\\ k_{-j} \end{matrix}\begin{matrix} \\ \text{A∙Ec∙S(ox)} \\ \end{matrix}$$

$$\begin{matrix} \\ \text{A∙Eo} \\ \end{matrix}\begin{matrix} k_{k} \\ \rightleftharpoons\\ k_{-k} \end{matrix}\begin{matrix} \\ \text{A∙Ec} \\ \end{matrix}$$

$$\begin{matrix} \\ \text{B∙Eo} \\ \end{matrix}\begin{matrix} k_{x} \\ \rightleftharpoons\\ k_{-x} \end{matrix}\begin{matrix} \\ \text{B∙Eo∙S(red)} \\ \end{matrix}\begin{matrix} k_{y} \\ \rightleftharpoons\\ k_{-y} \end{matrix}\begin{matrix} \\ \text{B∙Ec∙S(red)} \\ \end{matrix}$$

$$\begin{matrix} \\ \text{B∙Eo} \\ \end{matrix}\begin{matrix} k_{z} \\ \rightleftharpoons\\ k_{-z} \end{matrix}\begin{matrix} \\ \text{B∙Ec} \\ \end{matrix}$$

Combined, these lead to the following set of rate equations,

| $\frac{d}{dt}\left[ \text{A} \right]=k_{-1}\left[ \text{A∙Eo} \right]-k_{1}\left[ \text{A} \right]\left[ \text{E} \right]$ | (S1) |
| --- | --- |
| $\frac{d}{dt}\left[ \text{A∙Eo} \right]=k_{1}\left[ \text{A} \right]\left[ \text{E} \right]+k_{-k}\left[ \text{A∙Ec} \right]+k_{-2}\left[ \text{A∙Eo∙S(red)} \right]+k_{-i}\left[ \text{A∙Eo∙S(ox)} \right]-k_{k}\left[ \text{A∙Eo} \right]-k_{i}\left[ \text{A∙Eo} \right]\left[ \text{S(ox)} \right]-k_{-1}\left[ \text{A∙Eo} \right]-k_{2}\left[ \text{A∙Eo} \right]\left[ \text{S(red)} \right]$ | (S2) |
| $\frac{d}{dt}\left[ \text{A∙Eo∙S(red)} \right]=k_{2}\left[ \text{A∙Eo} \right]\left[ \text{S(red)} \right]+k_{-3}\left[ \text{A∙Ec∙S(red)} \right]{-k}_{-2}\left[ \text{A∙Eo∙S(red)} \right]{-k}_{3}\left[ \text{A∙Eo∙S(red)} \right]$ | (S3) |
| $\frac{d}{dt}\left[ \text{A∙Ec∙S(red)} \right]=k_{3}\left[ \text{A∙Eo∙S(red)} \right]+k_{-4}\left[ \text{B∙Ec∙S(ox)} \right]-k_{-3}\left[ \text{A∙Ec∙S(red)} \right]-k_{4}\left[ \text{A∙Ec∙S(red)} \right]$ | (S4) |
| $\frac{d}{dt}\left[ \text{B∙Ec∙S(ox)} \right]=k_{4}\left[ \text{A∙Ec∙S(red)} \right]+k_{-5}\left[ \text{B∙Eo∙S(ox)} \right]-k_{-4}\left[ \text{B∙Ec∙S(ox)} \right]-k_{5}\left[ \text{B∙Ec∙S(ox)} \right]$ | (S5) |
| $\frac{d}{dt}\left[ \text{B∙Eo∙S(ox)} \right]=k_{5}\left[ \text{B∙Ec∙S(ox)} \right]+k_{-6}\left[ \text{B∙Eo} \right]\left[ \text{S(ox)} \right]-k_{-5}\left[ \text{B∙Eo∙S(ox)} \right]-k_{6}\left[ \text{B∙Eo∙S(ox)} \right]$ | (S6) |
| $\frac{d}{dt}\left[ \text{B∙Eo} \right]=k_{6}\left[ \text{B∙Eo∙S(ox)} \right]+k_{-7}\left[ \text{B∙Eo} \right]+k_{-x}\left[ \text{B∙Eo∙S(red)} \right]++k_{-z}\left[ \text{B∙Ec} \right]-k_{-6}\left[ \text{B∙Eo} \right]\left[ \text{S(ox)} \right]-k_{7}\left[ \text{B∙Eo} \right]-k_{x}\left[ \text{B∙Eo} \right]\left[ \text{S(red)} \right]-k_{z}\left[ \text{B∙Eo} \right]$ | (S7) |
| $\frac{d}{dt}\left[ \text{B} \right]=k_{7}\left[ \text{B∙Eo} \right]-k_{-7}\left[ \text{B} \right]\left[ \text{E} \right]$ | (S8) |
| $\frac{d}{dt}\left[ \text{A∙Eo∙S(ox)} \right]=k_{i}\left[ \text{A∙Eo} \right]\left[ \text{S(ox)} \right]+k_{-j}\left[ \text{A∙Ec∙S(ox)} \right]-k_{-i}\left[ \text{A∙Eo∙S(ox)} \right]-k_{j}\left[ \text{A∙Eo∙S(ox)} \right]$ | (S9) |
| $\frac{d}{dt}\left[ \text{A∙Ec∙S(ox)} \right]=k_{j}\left[ \text{A∙Eo∙S(ox)} \right]-k_{-j}\left[ \text{A∙Ec∙S(ox)} \right]$ | (S10) |
| $\frac{d}{dt}\left[ \text{A∙Ec} \right]=k_{k}\left[ \text{A∙Eo} \right]-k_{-k}\left[ \text{A∙Ec} \right]$ | (S11) |
| $\frac{d}{dt}\left[ \text{B∙Eo∙S(red)} \right]=k_{x}\left[ \text{B∙Eo} \right]\left[ \text{S(red)} \right]+k_{-y}\left[ \text{B∙Ec∙S(red)} \right]-k_{-x}\left[ \text{B∙Eo∙S(red)} \right]-k_{y}\left[ \text{B∙Eo∙S(red)} \right]$ | (S12) |
| $\frac{d}{dt}\left[ \text{B∙Ec∙S(red)} \right]=k_{y}\left[ \text{B∙Eo∙S(red)} \right]-k_{-y}\left[ \text{B∙Ec∙S(red)} \right]$ | (S13) |
| $\frac{d}{dt}\left[ \text{B∙Ec} \right]=k_{z}\left[ \text{B∙Eo} \right]-k_{-z}\left[ \text{B∙Ec} \right]$ | (S14) |

We also assumed a constant concentration of available enzymes,

| $\left[ \text{E} \right]=\left[ \text{E}_{\text{total}} \right]-\left[ \text{A∙Eo} \right]-\left[ \text{A∙Ec} \right]-\left[ \text{A∙Eo∙S(ox)} \right]-\left[ \text{A∙Ec∙S(ox)} \right]-\left[ \text{A∙Eo∙S(red)} \right]-\left[ \text{A∙Ec∙S(red)} \right]-\left[ \text{B∙Eo} \right]-\left[ \text{B∙Ec} \right]-\left[ \text{B∙Eo∙S(ox)} \right]-\left[ \text{B∙Ec∙S(ox)} \right]-\left[ \text{B∙Eo∙S(red)} \right]-\left[ \text{B∙Ec∙S(red)} \right]$ | (S15) |
| --- | --- |

The system was driven into different equilibria by controlling $R$, the ratio of total product to total substrate, whose sum was fixed at value $T$,

| $R=\frac{\left[ \text{S(ox)}_{\text{total}} \right]}{\left[ \text{S(red)}_{\text{total}} \right]}$ | (S16) |
| --- | --- |
| $\left[ \text{T} \right]=\left[ \text{S(ox)}_{\text{total}} \right]+\left[ \text{S(red)}_{\text{total}} \right]$ | (S17) |
| $\left[ \text{S(ox)} \right]=\left[ \text{S(ox)}_{\text{total}} \right]-\left[ \text{A∙Eo∙S(ox)} \right]-\left[ \text{A∙Ec∙S(ox)} \right]-\left[ \text{B∙Eo∙S(ox)} \right]-\left[ \text{B∙Ec∙S(ox)} \right]$ | (S18) |
| $\left[ \text{S(red)} \right]=\left[ \text{S(red)}_{\text{total}} \right]-\left[ \text{A∙Eo∙S(red)} \right]-\left[ \text{A∙Ec∙S(red)} \right]-\left[ \text{B∙Eo∙S(red)} \right]-\left[ \text{B∙Ec∙S(red)} \right]$ | (S19) |

The total concentration of cofactors was also held constant,

| $\left[ \text{N} \right]=\left[ \text{A} \right]+\left[ \text{A∙Eo} \right]+\left[ \text{A∙Ec} \right]+\left[ \text{A∙Eo∙S(ox)} \right]+\left[ \text{A∙Ec∙S(ox)} \right]+\left[ \text{A∙Eo∙S(red)} \right]+\left[ \text{A∙Ec∙S(red)} \right]+\left[ \text{B} \right]+\left[ \text{B∙Eo} \right]+\left[ \text{B∙Ec} \right]+\left[ \text{B∙Eo∙S(ox)} \right]+\left[ \text{B∙Ec∙S(ox)} \right]+\left[ \text{B∙Eo∙S(red)} \right]+\left[ \text{B∙Ec∙S(red)} \right]$ | (S20) |
| --- | --- |

These equations were solved in MATLAB R2019a (The Mathworks, Cambridge, UK) using the fsolve() function and the parameter values in Table S4.

**Table S5:** Values of parameters used to solve the redox equilibrium model and their justifications

| **Parameter** | **Value** | **Justification** |
| --- | --- | --- |
| $k_{1}$ | 6.19 x 10^6^ M^-1^ s^-1^ | Measured by Zhadin et al.[5] |
| $k_{-1}$ | 559 s^-1^ |  |
| $k_{2}$ | 3.043 x 10^7^ M^-1^ s^-1^ |  |
| $k_{-2}$ | 1.05 x 10^5^ s^-1^ |  |
| $k_{3}$ | 940 s^-1^ |  |
| $k_{-3}$ | 470 s^-1^ |  |
| $k_{4}$ | 1350 s^-1^ |  |
| $k_{-4}$ | 1337 s^-1^ |  |
| $k_{5}$ | 210 s^-1^ |  |
| $k_{-5}$ | 595 s^-1^ |  |
| $k_{6}$ | 1750 s^-1^ |  |
| $k_{-6}$ | 2.01 x 10^7^ M^-1^ s^-1^ |  |
| $k_{7}$ | 90 s^-1^ |  |
| $k_{-7}$ | 5.6 x 10^7^ M^-1^ s^-1^ |  |
| $k_{i}$ | $k_{2}$ | Assuming the binding and unbinding of products and substrates are equally probable |
| $k_{-i}$ | $k_{-2}$ |  |
| $k_{j}$ | $k_{3}$ | Assuming the kinetics of the open/closed transition is the same with product or substrate |
| $k_{-j}$ | $k_{-3}$ |  |
| $k_{k}$ | $k_{z}$ | Assuming the ligand-free open/close kinetics are the same for NAD(P)+ or NAD(P)H bound |
| $k_{-k}$ | $k_{-z}$ |  |
| $k_{x}$ | $k_{-6}$ | Assuming the binding and unbinding of products and substrates are equally probable |
| $k_{-x}$ | $k_{6}$ |  |
| $k_{y}$ | $k_{-5}$ | Assuming the kinetics of the open/closed transition is the same with product or substrate |
| $k_{-y}$ | $k_{5}$ |  |
| $k_{z}$ | 41667 | Inverse of the 24µs time constant measured by Deng et al.[6] for binary complex closure. |
| $k_{-z}$ | 416667 | Derived from $k_{k}$and the 10% proportion of closed binary complexes measured in Blacker et al.[4] |
| $\left[ \text{E} \right]$ | 5 x 10^-6^ M | Chosen to give total bound NAD(P)H populations of ~10%, in line with values obtained using FLIM. This value is similar in magnitude to estimates published by Albe et al.[7] |
| $\left[ \text{T} \right]$ | 1 x 10^-3^ M | Order of magnitude estimate from Brooks et al.[8] |
| $\left[ \text{N} \right]$ | 500 x 10^-6^ M | From measurements by Zhu et al.[9] |

**Table S6:** Mean NAD(P)H fluorescence decay parameters in subcellular compartments of mammalian oocytes. Values in square brackets indicate mean standard deviation confidence intervals.

|  | **Mitochondria** | | | **Cytosol** | | | | **Nucleus** | | |
| --- | --- | --- | --- | --- | --- | --- | --- | --- | --- | --- |
| Control (n=17 cells) | | | | | | | | | | |
| $\tau_{1}$ / ps | 0.31 | | [0.3, 0.33] | 0.31 | [0.3, 0.32] | | | 0.32 | | [0.29, 0.35] |
| $\tau_{2}$ / ps | 1.20 | | [1.19, 1.21] | 1.16 | [1.15, 1.17] | | | 1.27 | | [1.22, 1.29] |
| $\tau_{3}$ / ps | 3.41 | | [3.38, 3.43] | 3.15 | [3.13, 3.17] | | | 3.53 | | [3.42, 3.59] |
| $a_{1}$ / % | 0.38 | | [0.37, 0.39] | 0.34 | [0.33, 0.35] | | | 0.39 | | [0.36, 0.42] |
| $a_{2}$ / % | 0.41 | | [0.4, 0.41] | 0.42 | [0.41, 0.42] | | | 0.41 | | [0.4, 0.42] |
| $a_{3}$ / % | 0.21 | | [0.21, 0.22] | 0.24 | [0.24, 0.24] | | | 0.20 | | [0.19, 0.2] |
| $\chi_{R}^{2}$ | 1.37 | | | 1.42 | | | | 1.09 | | |
| Pyruvate Only (n=10 cells) | | | | | | | | | | |
| $\tau_{1}$ / ps | 0.28 | [0.27, 0.29] | | 0.28 | | [0.27, 0.29] | | 0.26 | [0.22, 0.29] | |
| $\tau_{2}$ / ps | 1.00 | [0.99, 1.02] | | 0.98 | | [0.97, 1.00] | | 1.05 | [1.00, 1.10] | |
| $\tau_{3}$ / ps | 3.31 | [3.29, 3.34] | | 3.12 | | [3.09, 3.15] | | 3.65 | [3.41, 3.72] | |
| $a_{1}$ / % | 0.45 | [0.44, 0.46] | | 0.44 | | [0.43, 0.45] | | 0.46 | [0.41, 0.50] | |
| $a_{2}$ / % | 0.36 | [0.35, 0.36] | | 0.36 | | [0.36, 0.37] | | 0.38 | [0.36, 0.39] | |
| $a_{3}$ / % | 0.19 | [0.19, 0.19] | | 0.20 | | [0.19, 0.20] | | 0.16 | [0.16, 0.17] | |
| $\chi_{R}^{2}$ | 1.37 | | | 1.29 | | | | 1.06 | | |
| Lactate Only (n=11 cells) | | | | | | | | | | |
| $\tau_{1}$ / ps | 0.39 | [0.38, 0.4] | | 0.38 | | | [0.37, 0.39] | 0.34 | [0.32, 0.36] | |
| $\tau_{2}$ / ps | 1.28 | [1.28, 1.29] | | 1.22 | | | [1.21, 1.23] | 1.15 | [1.13, 1.16] | |
| $\tau_{3}$ / ps | 3.02 | [3.01, 3.04] | | 2.80 | | | [2.79, 2.81] | 2.77 | [2.75, 2.8] | |
| $a_{1}$ / % | 0.33 | [0.33, 0.34] | | 0.30 | | | [0.29, 0.31] | 0.30 | [0.29, 0.32] | |
| $a_{2}$ / % | 0.44 | [0.44, 0.45] | | 0.44 | | | [0.44, 0.45] | 0.43 | [0.43, 0.44] | |
| $a_{3}$ / % | 0.22 | [0.22, 0.23] | | 0.26 | | | [0.26, 0.26] | 0.26 | [0.26, 0.27] | |
| $\chi_{R}^{2}$ | 2.22 | | | 2.26 | | | | 1.23 | | |

**Table S7:** Mean NAD(P)H fluorescence decay parameters in mammalian oocytes with compartmentalised lifetimes shared between conditions. Values in square brackets indicate mean standard deviation confidence intervals.

|  | **Mitochondria** | | | **Cytosol** | | | | **Nucleus** | | |
| --- | --- | --- | --- | --- | --- | --- | --- | --- | --- | --- |
| $\tau_{1}$ / ps | 0.18 | | [0.17, 0.19] | 0.18 | [0.17, 0.19] | | | 0.17 | | [0.14, 0.19] |
| $\tau_{2}$ / ps | 0.55 | | [0.54, 0.56] | 0.57 | [0.56, 0.58] | | | 0.57 | | [0.55, 0.58] |
| $\tau_{3}$ / ps | 1.67 | | [1.66, 1.67] | 1.66 | [1.65, 1.67] | | | 1.64 | | [1.63, 1.65] |
| $\tau_{4}$ / ps | 4.05 | | [4.03, 4.07] | 3.83 | [3.81, 3.85] | | | 3.69 | | [3.65, 3.74] |
| Control (n=17 cells) | | | | | | | | | | |
| $a_{1}$ / % | 0.2 | | [0.13, 0.26] | 0.19 | [0.13, 0.25] | | | 0.21 | | [0.05, 0.38] |
| $a_{2}$ / % | 0.33 | | [0.3, 0.37] | 0.31 | [0.28, 0.34] | | | 0.33 | | [0.24, 0.41] |
| $a_{3}$ / % | 0.35 | | [0.33, 0.36] | 0.37 | [0.35, 0.38] | | | 0.33 | | [0.3, 0.37] |
| $a_{4}$ / % | 0.12 | | [0.12, 0.13] | 0.13 | [0.12, 0.13] | | | 0.13 | | [0.11, 0.15] |
| Pyruvate Only (n=10 cells) | | | | | | | | | | |
| $a_{1}$ / % | 0.29 | [0.21, 0.36] | | 0.31 | | [0.23, 0.38] | | 0.34 | [0.1, 0.58] | |
| $a_{2}$ / % | 0.37 | [0.33, 0.41] | | 0.36 | | [0.32, 0.4] | | 0.34 | [0.23, 0.46] | |
| $a_{3}$ / % | 0.23 | [0.22, 0.25] | | 0.23 | | [0.21, 0.25] | | 0.22 | [0.17, 0.27] | |
| $a_{4}$ / % | 0.11 | [0.1, 0.12] | | 0.11 | | [0.1, 0.11] | | 0.1 | [0.08, 0.13] | |
| Lactate Only (n=11 cells) | | | | | | | | | | |
| $a_{1}$ / % | 0.09 | [0.05, 0.13] | | 0.09 | | | [0.06, 0.13] | 0.11 | [0.04, 0.18] | |
| $a_{2}$ / % | 0.35 | [0.33, 0.37] | | 0.34 | | | [0.32, 0.36] | 0.34 | [0.31, 0.38] | |
| $a_{3}$ / % | 0.46 | [0.45, 0.46] | | 0.46 | | | [0.46, 0.47] | 0.44 | [0.42, 0.46] | |
| $a_{4}$ / % | 0.1 | [0.1, 0.11] | | 0.1 | | | [0.1, 0.11] | 0.11 | [0.1, 0.11] | |
| $\chi_{R}^{2}$ | 1.64 | | | 1.68 | | | | 1.13 | | |


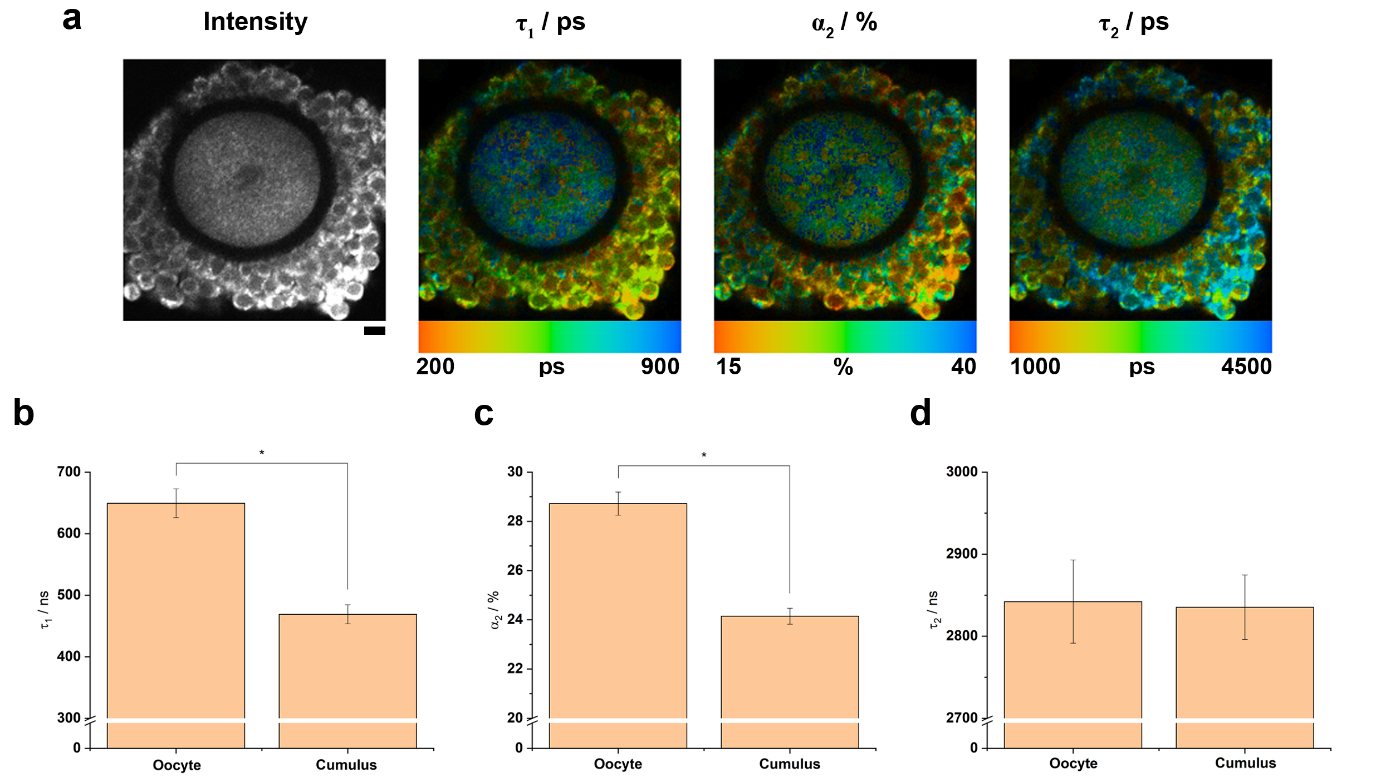


**Figure S3:** (a) Representative NAD(P)H FLIM images of mammalian oocytes with intact cumulus cells (scale bar 10µm). (b-d) Fluorescence decay parameters τ_1_, α_2_, and τ_2_ from pixel-by-pixel biexponential fitting averaged across n=13 separate preparations. Error bars are ±SEM.


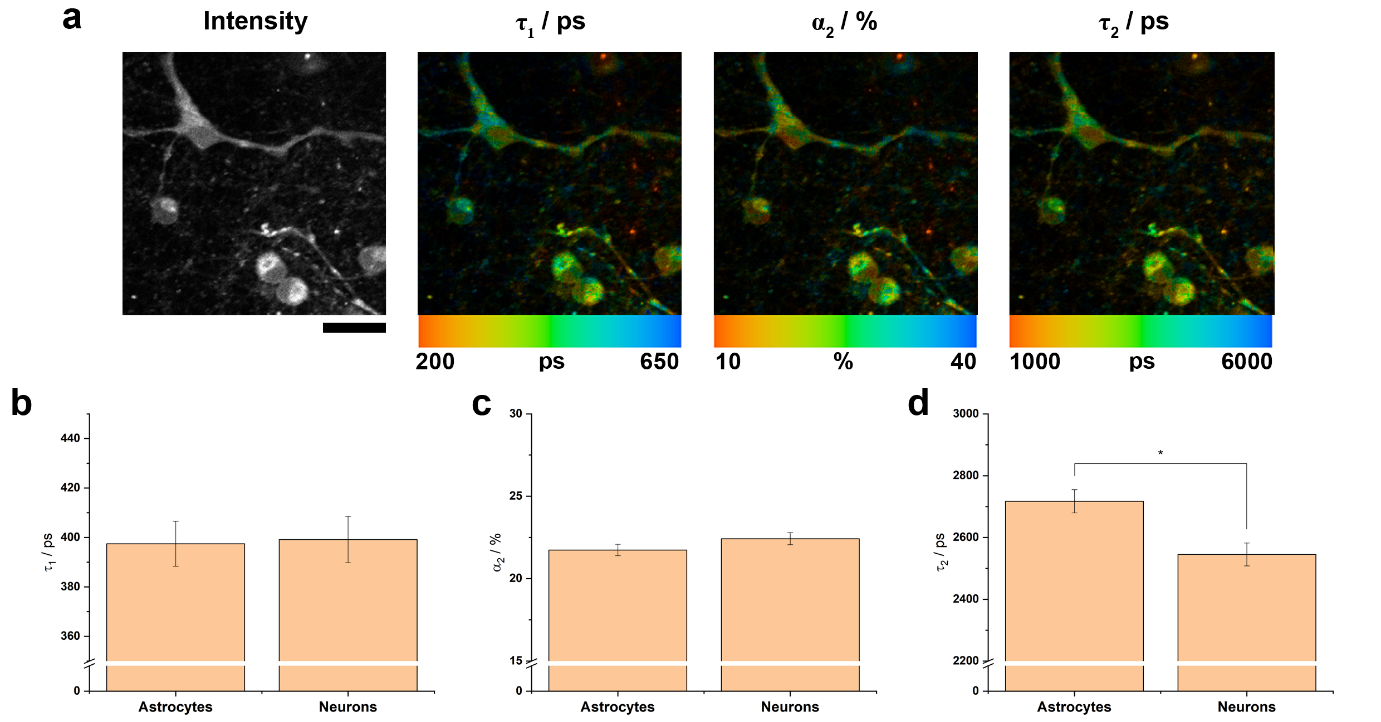


**Figure S4:** (a) Representative NAD(P)H FLIM images of mixed co-cultures of cortical neurons and astrocytes (scale bar 100µm). (b-d) Fluorescence decay parameters τ_1_, α_2_, and τ_2_ from pixel-by-pixel biexponential fitting averaged across n=31 images from 8 separate coverslips. Error bars are ±SEM.


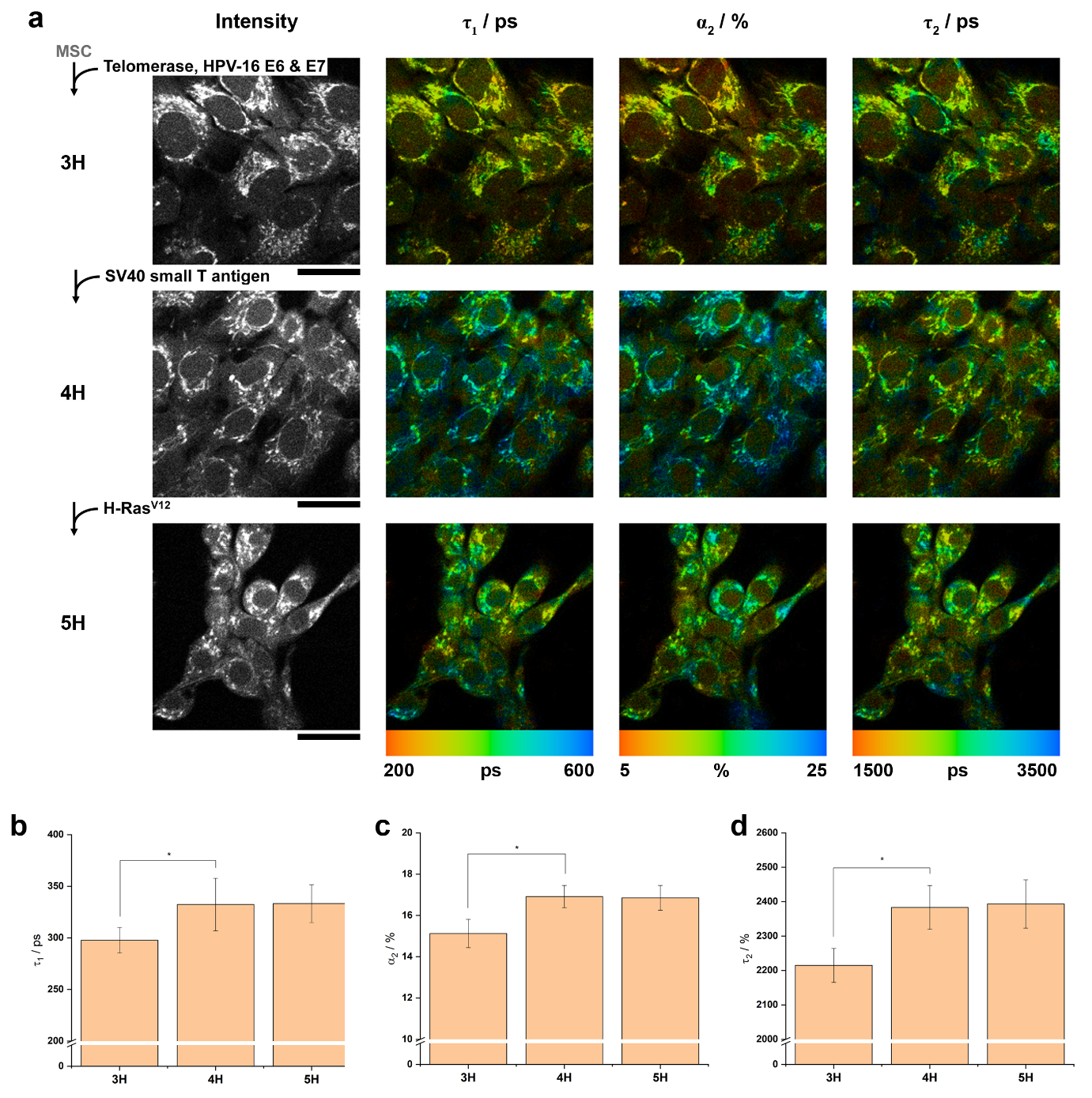


**Figure S5:** (a) Representative NAD(P)H FLIM images of a mesenchymal stem cell model of oncogenesis[10] (scale bar 100µm). (b-d) Fluorescence decay parameters τ_1_, α_2_, and τ_2_ from pixel-by-pixel biexponential fitting averaged across n=21 images from 6 separate coverslips (3H), n=22 images from 7 coverslips (4H) and n=18 images from 7 coverslips (5H). Error bars are ±SEM.

**Table S8:** Metabolic characterisation of transformed mesenchymal stem cells. Oxygen consumption experiments were repeated n=8 (3H), n=14 (4H) and n=11 (5H) times. Extracellular acidification experiments were repeated 5 times for each cell type. Uncertainties are ±SEM.

|  | **3H** | **4H** | **5H** |
| --- | --- | --- | --- |
| Oxygen Consumption / 10^-17^ moles s^-1^ cell^-1^ | | | |
| Routine | 1.6(±0.2) | 2.4(±0.5) | 2.4(±0.5) |
| Leak | 0.49(±0.06) | 0.9(±0.4) | 0.5(±0.1) |
| ETS | 2.1(±0.2) | 1.7(±0.1) | 3.3(±0.6) |
| ATP-linked | 1.1(±0.2) | 1.5(±0.2) | 1.9(±0.4) |
| Extracellular Acidification / mpH min^-1^ | | | |
| Resting Glycolysis | 23(±2) | 28(±4) | 22(±1) |
| Maximum Capacity | 35(±3) | 58(±8) | 33(±2) |

**Appendix S2:** Comparisons with an existing coarse-grained model of NAD(P)H FLIM

To successfully relate NAD(P)H FLIM measurements to metabolic flux, the model developed by Yang et al. included the assumption that NAD(P)H bound to “reductases”, which they define as enzymes that reduce NAD(P)+ to NAD(P)H, exhibits a different fluorescence lifetime than that bound to “oxidases”, defined as enzymes that oxidise NADH to NAD+[11]. We here show that their model and calibration lead to the conclusion that the reductase lifetime $\tau_{\text{red}}$ is larger than the oxidase lifetime $\tau_{\text{ox}}$, a finding supported by the conclusions of our work.

Yang et. al define two experimentally determined parameters that relate $\tau_{\text{red}}$ and $\tau_{\text{ox}}$ to the distinct rates of NAD(P)H binding ($k_{\text{ox}}^{\text{b}}$ and $k_{\text{red}}^{\text{b}}$) and unbinding ($k_{\text{ox}}^{\text{u}}$ and $k_{\text{red}}^{\text{u}}$) to an oxidase or reductase,

| $A=\left( \tau_{\text{ox}}-\tau_{\text{red}} \right)\frac{k_{\text{ox}}^{\text{b}}+k_{\text{red}}^{\text{b}}}{k_{\text{ox}}^{\text{u}}-k_{\text{red}}^{\text{u}}}$ | (S21) |
| --- | --- |
| $B=\frac{k_{\text{ox}}^{\text{u}}\tau_{\text{red}}-k_{\text{red}}^{\text{u}}\tau_{\text{ox}}}{k_{\text{ox}}^{\text{u}}-k_{\text{red}}^{\text{u}}}$ | (S22) |

For convenience, we eliminate all unbinding constants using the corresponding dissociation constants,

| $A=\left( \tau_{\text{ox}}-\tau_{\text{red}} \right)\frac{k_{\text{ox}}^{\text{b}}+k_{\text{red}}^{\text{b}}}{K_{D}^{\text{ox}}k_{\text{ox}}^{\text{b}}-{K_{D}^{\text{red}}k}_{\text{red}}^{\text{b}}}$ | (S23) |
| --- | --- |
| $B=\frac{K_{D}^{\text{ox}}k_{\text{ox}}^{\text{b}}\tau_{\text{red}}-{K_{D}^{\text{red}}k}_{\text{red}}^{\text{b}}\tau_{\text{ox}}}{K_{D}^{\text{ox}}k_{\text{ox}}^{\text{b}}-{K_{D}^{\text{red}}k}_{\text{red}}^{\text{b}}}$ | (S24) |

Dividing these leads to,

| $\frac{\tau_{\text{ox}}}{\tau_{\text{red}}}=\frac{1+\frac{k_{\text{red}}^{\text{b}}}{k_{\text{ox}}^{\text{b}}}\left( 1-\frac{A}{B}K_{D}^{\text{red}} \right)}{1+\frac{k_{\text{red}}^{\text{b}}}{k_{\text{ox}}^{\text{b}}}+\frac{A}{B}K_{D}^{\text{ox}}}$ | (S25) |
| --- | --- |

Yang et al. determine $A/B$ as ${0.3}/{1.6=0.19}$. Stinson et al. measured $K_{D}^{\text{red}}\sim$3.5µM for the “reductase” type LDH1 found in heart and $K_{D}^{\text{ox}}\sim$0.5µM for the “oxidase” type LDH5 found in muscle. Substituting these values allows us to plot the ratio of lifetimes against the ratio of binding rates (Figure S4) where it can be seen that $\tau_{\text{red}}>\tau_{\text{ox}}$.

**Figure S6:** A plot of Equation S25 for experimentally determined parameter values.

**Supporting References**

1 Fisz JJ (2007) Another Look at Magic-Angle-Detected Fluorescence and Emission Anisotropy Decays in Fluorescence Microscopy. J Phys Chem A 111, 12867–12870.

2 Fisz JJ (2007) Fluorescence Polarization Spectroscopy at Combined High-Aperture Excitation and Detection:  Application to One-Photon-Excitation Fluorescence Microscopy. J Phys Chem A 111, 8606–8621.

3 Blacker TS, Nicolaou N, Duchen MR & Bain AJ (2019) Polarized Two-Photon Absorption and Heterogeneous Fluorescence Dynamics in NAD(P)H. J Phys Chem B 123, 4705–4717.

4 Blacker TS, Duchen MR & Bain AJ (2023) NAD(P)H binding configurations revealed by time-resolved fluorescence and two-photon absorption. Biophys J 122, 1240–1253.

5 Zhadin N, Gulotta M & Callender R (2008) Probing the Role of Dynamics in Hydride Transfer Catalyzed by Lactate Dehydrogenase. Biophys J 95, 1974–1984.

6 Deng H, Zhadin N & Callender R (2001) Dynamics of Protein Ligand Binding on Multiple Time Scales:  NADH Binding to Lactate Dehydrogenase. Biochemistry 40, 3767–3773.

7 Albe KR, Butler MH & Wright BE (1990) Cellular concentrations of enzymes and their substrates. J Theor Biol 143, 163–195.

8 Brooks GA (2020) Lactate as a fulcrum of metabolism. Redox Biol 35, 101454.

9 Zhu X-H, Lu M, Lee B-Y, Ugurbil K & Chen W (2015) In vivo NAD assay reveals the intracellular NAD contents and redox state in healthy human brain and their age dependences. Proc Natl Acad Sci 112, 2876–2881.

10 Funes JM, Quintero M, Henderson S, Martinez D, Qureshi U, Westwood C, Clements MO, Bourboulia D, Pedley RB, Moncada S & Boshoff C (2007) Transformation of human mesenchymal stem cells increases their dependency on oxidative phosphorylation for energy production. Proc Natl Acad Sci 104, 6223–6228.

11 Yang X, Ha G & Needleman DJ (2021) A coarse-grained NADH redox model enables inference of subcellular metabolic fluxes from fluorescence lifetime imaging. eLife 10, e73808.
